# Distinct neural mechanisms for heading retrieval and context recognition in the hippocampus during spatial reorientation

**DOI:** 10.21203/rs.3.rs-2724785/v1

**Published:** 2023-03-31

**Authors:** Celia M. Gagliardi, Marc E. Normandin, Alexandra T. Keinath, Joshua B. Julian, Matthew R. Lopez, Manuel-Miguel Ramos-Alvarez, Russell A. Epstein, Isabel A. Muzzio

**Affiliations:** 1 Department of Neuroscience, Development, and Regenerative Biology, University of Texas at San Antonio, 1 UTSA Circle, San Antonio, TX 78249, USA; 2 Department of Psychiatry, Douglas Hospital Research Centre, McGill University, 6875 Boulevard LaSalle, Verdun, QC, H4H 1RS, Canada; 3 Princeton Neuroscience Institute, Princeton University, Princeton, NJ, USA; 4 Psychology Department, University of Jaen, Campus Las Lagunillas, Jaen 23071, Spain; 5 Department of Psychology, University of Pennsylvania, Philadelphia, PA 19104, USA; 6 Department of Psychological & Brain Sciences, University of Iowa, Iowa City, IA 52245, USA

**Keywords:** spatial memory, reorientation, learning, navigation, hippocampus, place cells

## Abstract

Reorientation, the process of regaining one’s bearings after becoming lost, requires identification of a spatial context (context recognition) and recovery of heading direction within that context (heading retrieval). We previously showed that these processes rely on the use of features and geometry, respectively. Here, we examine reorientation behavior in a task that creates contextual ambiguity over a long timescale to demonstrate that mice learn to combine both featural and geometric cues to recover heading with experience. At the neural level, most CA1 neurons persistently align to geometry, and this alignment predicts heading behavior. However, a small subset of cells shows feature-sensitive place field remapping, which serves to predict context. Efficient heading retrieval and context recognition require integration of featural and geometric information in the active network through rate changes. These data illustrate how context recognition and heading retrieval are coded in CA1 and how these processes change with experience.

## Introduction

When navigating organisms lose their bearings, their internal sense of direction is unreliable, and they must use external cues to identify the surroundings and regain their sense of direction within that context. This process, known as spatial reorientation, has been studied extensively in the animal behavior and developmental psychology literatures (for review, see Cheng & Newcombe, 2005). However, with some exceptions (Keinath et al., 2017; Weiss et al., 2017), the neural mechanisms that underlie reorientation are largely unknown. The current study attempts to address this lacuna, with particular emphasis on understanding how different kinds of external cues are used for spatial reorientation, and how they might support different components of the reorientation task.

Previous behavioral findings across many species show that the geometry of the environment — the shape of the layout — plays a predominant role in reorientation (Cheng, 1986; Julian et al., 2015; Hermer & Spelke, 1996; Lee et al., 2012). This is demonstrated by the inability of disoriented subjects to discriminate between geometrically equivalent locations in symmetrical environments (e.g., opposite corners of a rectangular chamber), even when these locations could be disambiguated by non-geometric featural cues such as colors, sounds, and textures (Cheng, 1986; Cheng et al., 2013; Sovrano et al., 2012; Vallortigara et al., 2004). Several theories of reorientation focused on explaining the cognitive mechanisms underlying this pattern of behavior. View matching theory suggests that navigators identify targets by matching the current panoramic view of a location with a stored representation of the goal area (Collett & Collett, 2002; Sheynikhovich et al., 2009; Sturzl et al., 2008), a process that relies on visual recognition rather than retrieval of a cognitive map of the layout. This theory can account for geometric errors and has gained support from research with ants and chicks (Pecchia & Vallortigara, 2010b, 2010a; Wystrach et al., 2011), but it has been challenged by findings showing that children can reorient using geometry regardless of the contextual viewpoint (Huttenlocher & Vasilyeva, 2003) and that visually impaired subjects can reorient using geometry (Landau et al., 1981; Normandin et al., 2022; Sovrano et al., 2018). The geometric module view, on the other hand, proposes that navigators reorient based solely on a cognitive representation of environmental geometry that is impermeable to non-geometric information (Gallistel, 1990; Hermer & Spelke, 1996; Lee & Spelke, 2010a, 2010b). This theory gained substantial support from behavioral studies in various animal models (Lee & Spelke, 2010a; Vallortigara, 2009), as well as neurophysiological recordings in rodents (Lee & Spelke, 2010a; Keinath et al., 2017; Vallortigara, 2009). However, it has been challenged by studies showing that navigators sometimes use non-geometric (“featural”) information when reorienting, especially after having experience with features (Bodily et al., 2013; Ratliff & Newcombe, 2008; Twyman et al., 2007). These data indicate that non-geometric information is not completely ignored, though its use may depend on situational factors (Cheng et al., 2013; Lee & Spelke, 2010b; Newcombe & Huttenlocher, 2006; Lee et al., 2020).

To account for the use of non-geometric information during reorientation, supporters of the geometric module view have suggested that reorientation relies on two independent processes, one relying on a modular representation of the shape of the layout and a second one involving associations of features and goal locations through reinforcement learning (Lee et al., 2006; Lee & Spelke, 2010b). This two-process account has, in turn, been questioned by critics who argued that the use of featural information can occur without a second process of associative learning when features are salient or have strong predictive directional value (Newcombe et al., 2010). These ideas were developed in two more recent theories: 1) The adaptive cue combination theory, which proposes that navigators combine or select cues using probabilistic approaches that weight their salience and reliability (Newcombe & Huttenlocher, 2006; Xu et al., 2017) and 2) The associative learning model, which suggests that geometric or featural cues gain or lose asssociative strength by adjusting their salience (Miller, 2009; Miller & Shettleworth, 2007). According to these theories, disoriented subjects reorient relying on only one process that uses geometry, features, or a combination of both depending on which cue or combination is the most informative.

How can these theoretical disputes be resolved? One potential limiting factor of previous studies is that they have almost universally tested reorientation in a single experimental chamber whose identity never varies. In real-world scenarios, however, lost navigator must accomplish *two* cognitive processes to reorient: 1) identify the environment they are in (*context recognition*), and 2) recover facing direction *(heading retrieval)* within that environment. Because most studies only use a single chamber, they only examine the second process. In a previous study, we used a novel two-context reorientation paradigm to show that disoriented mice use geometry for heading retrieval but non-geometric features for context recognition (Julian et al., 2015). This pattern or results suggests that heading retrieval and context recogntion are cognitively dissociable; moreover, they suggest the intriguing possibility that geometry and non-geometric features may make differential contributions to these different components of reorientation. However, this purely behavioral study did not probe the neural mechanisms underlying these processes. Furthermore, it did not investigate the possibility that as animals attend to the features for context recognition, they might also learn their directional value and incorporate featural information for heading retrieval with experience, a finding that would challenge the modularity of geometric representations.

We began to address the neural basis of one reorientation component—heading retrieval—in a previous study that examined the firing patterns of hippocampal place cells when disoriented mice reoriented in a classical single-context reorientation paradigm (Keinath et al., 2017). Place cells selectively fire when a navigator occupies specific locations in an environment (O’Keefe & Dostrovsky, 1971). In oriented animals, the place cells’ firing fields can be modulated by both geometry (O’Keefe & Burgess, 1996) and features (Muller & Kubie, 1987). Moreover, when animals are placed in different environments that are distinguishable in terms of features or geometry, alterations are observed in firing rate (rate remapping: Huxter et al., 2003), and/or firing location (location remapping: Colgin et al., 2008; Muller & Kubie, 1987). This allows place cells to represent both the location of the navigator within an environment and the identity of the environment itself, and the information underlying these computations can be both geometric and featural (Bostock et al., 1991; Lever et al., 2002). In our previous study, we found that the place field map of disoriented animals exhibited two possible orientations, consistent with the geometric ambiguity of the rectangular chamber, despite the presence of a disambiguating featural cue. Moreover, this geometry-based alignment served to predict reorientation behavior, suggesting that the hippocampal alignment subserved heading retrieval. Similar results have been observed in head direction cells and entorhinal grid cells (Weiss et al., 2017). However, since these studies only used one experimental chamber, they did not address the crucial question of how neural systems simultaneously implement context recognition and heading retrieval in situations of contextual ambiguity and whether neural representations change when animals learn the directional value on non-geometric cues with experience.

In the current study, we attempted to close this knowledge gap by recording from hippocampal area CA1 while mice performed an extended version of the two-context reorientation task. We focused on CA1 because different place cell subpopulations within this region have been shown to be involved in detecting distinct aspects of contextual change (Shin et al., 2022), a characteristic that would be essential to simultaneously code context recognition and heading retrieval. First, we investigated if geometry-based heading retrieval persisted over time in a paradigm where animals were attending to non-geometric features for context recognition. To this end, we modified the two-context behavioral paradigm so that training extended over a longer period than in our previous study (Julian et al., 2015). Second, we investigated how context recognition and heading retrieval were implemented in the hippocampus—in particular, whether they were mediated by dissociable neural processes as suggested by the behavior—and how these processes might change as animals became more familiar with the task. To address these questions, we conducted electrophysiological recordings and calcium imaging to characterize changes at the single cell and population level and performed longitudinal analysis of temporal dynamics on the active network during reorientation. Our results provide strong support for geometry-based reorientation and the existence of a separate cognitive process underlying feature/reward associations; however, they also demonstrate that the cognitive map that guides reorientation is not modular and that navigators can adapt their strategies based on the predicted directional value of available cues.

## Results

### Reorienting behavior in a two-context paradigm

**Figure 1.**
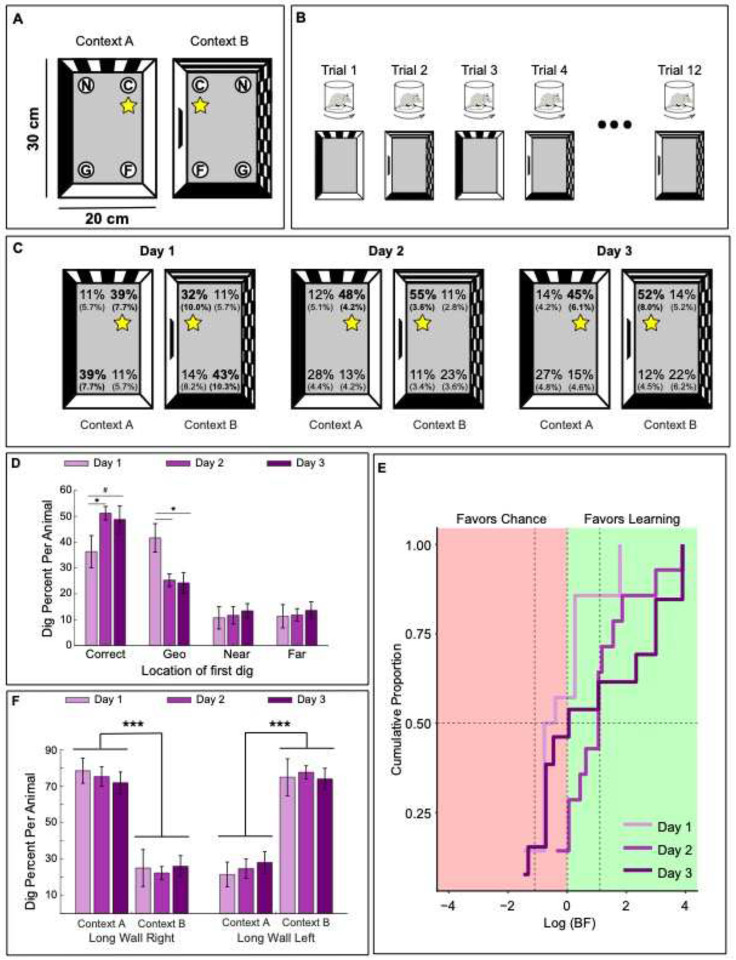
Reorientation behavior in a two-context paradigm. A) Schematic of experimental settings showing reward location (yellow star) in Context A (left) and Context B (right). B) Session structure of two context paradigm. Animals (N = 14) are disoriented before being placed in each context, in alternating order. C) Percentage of digs in each cup location on test trials on day 1 (left), day 2 (center), and day 3 (right). D) Histograms showing percentage of digs in each cup location combining both contexts. E. Cumulative proportion of individual Bayes Factors (BF) across days (1 to 3). Conventional values showing the border marking credibility for Malt (log(BF) > log(1/3)= 1.1) and Mnull (log(BF) < log(1/3)= −1.1) are indicated by vertical dashed lines. The value of half (0.5) of the sample is marked by a horizontal dashed line. F. Histogram showing percentage of digs in geometrically correct vs. incorrect axes in each context. Asterisks (*) represent significance using alpha = 0.05. Error bars denote ± 1 standard error of the mean (SEM)

To investigate heading retrieval and context recognition, we trained 14 disoriented male mice in a novel two-context reorientation paradigm to find a reward hidden in one out of four cups embedded in each corner of two chambers of identical rectangular shape (contexts). The contexts were distinguished by distinct polarizing features and reward locations (Figure 1A–B). Figure 1C represents the average percentage of first digs in each of the four cup locations in each context (C: correct rewarded location, G: geometric equivalent location to C, N: near location adjacent to the same short wall as C, F: far location adjacent to the same long wall as C; 4 test trials on Day 1, 12 test trials on Days 2 and 3). On day 1, animals searched more often in the geometrically appropriate corners (C and G) than geometrically inappropriate corners (N and F) in each chamber (Figure 1C, left panel), displaying a dissociable pattern of digging behavior that distinguished between the two contexts. These results corroborate our previous findings showing that mice initially use the geometry of the layout for heading retrieval, evident in the similar number of digs in geometrically equivalent cup locations (Julian et al., 2015). Notably, additional training in the task served to modify the reorientation strategies, which was evident in more digs in the rewarded corner (C) and fewer digs in the geometric corner (G) on days 2 and 3 (Figure 1C, days 2 and 3). This behavioral pattern suggests that animals eventually use features to distinguish geometrically equivalent corners, in addition to using them to disambiguate the contexts.

To confirm the shift in reorientation strategies, we conducted a repeated measures ANOVA on the combined dig locations in both contexts, with day of testing (day 1 to 3) and digging locations (C, G, N, and F) as within subjects’ factors (Figure 1D, note that these locations are different in the two contexts but are combined in this analysis). Results revealed a main effect of dig location [*F*(2.18, 28.36) = 29.30, *p* < .0001], as well as an interaction between day and dig location [*F*(4.35, 56.53) =2.60, *p* = .042], but no effect of day [*F*<1]. Post hoc analyses using Rom’s multiple comparisons revealed a difference in first dig locations across days. On day 1, digs in the correct and geometrically equivalent locations were higher than in the incorrect ones (C-N, C-F, G-N, and G-F, p< .05), with no difference between C and G (p> .05), or N and F (p> .05). However, on days 2 and 3, digs in C were significantly higher than digs in other locations (C-G, C-N, and C-F, p< .05), with no differences between all other locations (p> .05). Moreover, digs in the correct location were significantly higher on day 2 in comparisons to day 1 (C: Day 1 vs Day 2, p< .05) and displayed a trend on day 3 in comparison to day 1 (C: Day 1 vs Day 3, p= .07), while digs in the G location decreased on days 2 and 3 in comparison to day 1 (G: Day 1 vs Day 2 and Day 1 vs Day 3, *p*< .05). These data indicate that, over time, animals incorporate features to recover heading direction.

In order to consider the individual learning of each mouse, we also used hierarchical Bayesian analysis to test the alternative model (M_Alt_) that animals dug in the rewarded cup location vs. the null model (M_null_) that animals dug by chance [log(BF)> 1.1 provides credibility for M_Alt_, log(BF)< −1.1 provides credibility for M_null_]. The cumulative distributions of individual BF and global BF analysis provided credibility for M_Alt_ only on days 2 and 3 (log(BF)> 1.1), indicating that animals dug in C more than the others locations (N, G, or F), supporting the conclusion that mice incorporate the use of features for heading retrieval with experience [Figure 1E, group BFs: day 1: log(BF)= −2.61; day 2: log(BF)= 15.058; day 3: log(BF) = 17.67, for details on individual data see Table S1].

The analyses above illustrated the behavioral strategies that animals used to recover heading independent of context. To corroborate that mice distinguished the contexts by showing distinct digging behavior in each context, we calculated the proportion of digs in the geometrically correct and incorrect axes in each chamber (i.e., these axes will be opposite in each context) and conducted a 3 way repeated measures ANOVA using absolute corner location (long wall right or left), context (A or B), and day of testing (day 1, 2, or 3) as within subjects’ factors (Figure 1F). The results only showed a significant interaction between absolute corner location and context [*F*(1.00, 13.00) = 96.55, *p* < .0001]. Post hoc analyses using Rom’s multiple comparisons revealed that percentage of digs on the axis defined by long wall right (75.3%) were higher than digs along the axis defined by long wall left (24.7%) in Context A (p <.05), but the opposite occurred in Context B (long wall right: 24.4% vs. long wall left: 75.6%, p <.05). These results demonstrate that animals distinguish the contexts using their unique featural cues, which is evident in the distinct digging patterns in each chamber. We corroborated these results using BF evaluating the alternative model (M_Alt_) that animals preferentially dug on the rewarded axis in each context (long wall right in Context A and long wall left in Context B) vs. the null model (M_null_) that animals dug by chance (Figure S1, Table S2). In summary, animals initially rely on geometry for heading retrieval and features for context recognition, but ultimately also incorporate features to recover heading and disambiguate geometrically equivalent locations to maximize reward.

### Place field alignment to spatial geometry persists over days

Our previous work on reorientation in single contexts showed that pyramidal cells in CA1 align to the geometry of the environmental layout by remapping to symmetrical locations within the chamber. This occurred even in the presence of directionally informative non-geometric cues, thus providing a potential neural mechanism for heading retrieval (Keinath et al., 2017). With this previous result in mind, we tested if hippocampal alignment to geometry persisted over time when animals performed a two-context reorientation task that required attention to non-geometric features for context recognition. In this section we analyzed the relationship between the hippocampal map and geometry without consideration of possible differences between the contexts (that its, treating both contexts as if they were the same). In sections below, we analyze potential contextual contributions.

**Figure 2.**
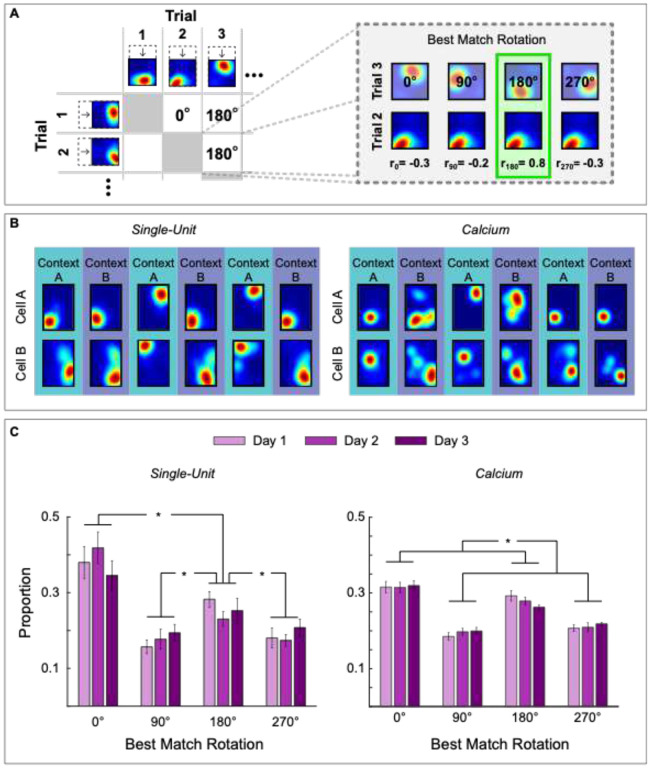
Place field alignment to spatial geometry persists over days. A) Quantification of best match rotation between trial maps for a cell. Place cells’ maps are compared across all trials to determine which rotation yields the highest correlation between each pair of maps. B) Example place cell maps from two simultaneously recorded cells on day 3 from single-unit (left) and calcium-imaging (right) data. C) Distribution of best-match rotations across days using single-unit recordings (*N* = 6, left) and calcium-imaging (*N* = 5, right), computed as the proportion of pairwise trial comparisons for which each rotation yielded the best match. Asterisks (*) represent significance using alpha = 0.05. Error bars denote ± 1 SEM.

Geometric alignment was evaluated using a best match rotation analysis, as previously described (Keinath et al., 2017). In this analysis, each cell’s place map was compressed to a square and compared across trials (66 pairwise comparisons for 12 trials, see STAR Methods for details). For each pairwise comparison, the map of Trial A was rotated 0°, 90°, 180°, and 270° and the pixel-to-pixel cross-correlation to the non-rotated map of Trial B was calculated to evaluate the similarity between the maps. The rotation that yielded the highest pixel-to-pixel cross correlation between trials was selected as the best match rotation (Figure 2A). The proportion of best match rotations for all cells was averaged for each animal on each day. Since rectangular chambers have 2 orders of rotational symmetry, high best match rotations values at 0° and 180° indicate place field alignment to geometry.

We analyzed the proportion of best match rotations using 3 × 4 repeated measures ANOVAs (days: 1, 2, and 3; Rotation: 0°, 90°, 180°, and 270°; both as within subjects’ factors), performed separately on maps generated using single-units (7 animals, Figure 2B, left) or (5 animals, Figure 2B, right). The results from both datasets were consistent. There were no main effects of day (Fs<1), or interactions between day and rotation (Fs<1), but significant main effects of rotation, in place cell maps generated from single units [Figure 2C, left panel; *F*(1.1,5.5) = 20.78, p= .0040] and calcium-transients [Figure 2C, right panel; *F*(1.18,4.74) = 71.48, p< .0001]. These results indicate that not all rotations yielded the best match equally often. Post hoc Rom’s tests showed that the best match rotations at 0° and 180° occurred more often than other rotations (p< .05) for both single-unit and calcium transient maps, with no differences between 90° and 270° rotations (p> .05). We corroborated these findings using an alternative procedure that calculated the angle from the center of the rectangular map to the center-of-mass of the place field. This analysis also yielded maximal alignment to 0° and 180° between all pairwise comparisons (Figure S2), indicating that geometric alignment was robust and did not depend on the quantification method. Finally, the proportion of best match rotations calculated using single-unit recordings revealed that no rotation (0°) occurred more often than 180° rotations (p< .05). This difference is also evident in the alignment of calcium trace maps on day 3 in the analysis per context (see below). These data replicate our previous results showing that the hippocampal map aligns to geometry in disoriented mice (Keinath et al., 2017) and further demonstrates that the alignment persists even after animals incorporate non-geometric features to recover heading.

### Different CA1 cells display distinct location remapping across contexts

**Figure 3.**
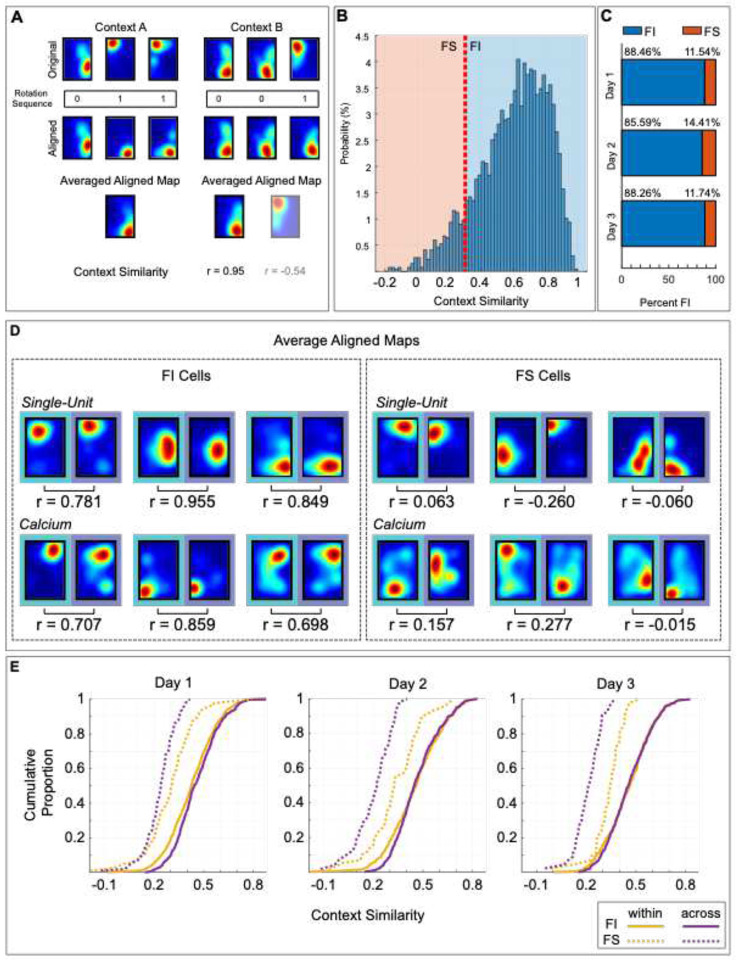
Different CA1 cells display distinct location remapping across contexts. A) Schematic of map alignment procedure. Since place fields in disoriented animals align to the geometry of the chamber, maps are first aligned according to the orientation (0° or 180° rotation) that yields the highest correlation across trials in each chamber, creating a binary sequence that illustrates whether the map remains stable (0) or rotates 180° (1) to produce the maximal similarity. Then, an average map of each aligned context is calculated, and the average maps are aligned relative to each other. B) Across context distribution of correlations between average aligned maps (context similarity) for all cells (*n* = 2669). The distribution shows a strong leftward skewness, indicating that although spatial maps are highly similar across contexts for most place cells, a moderate proportion of cells remap across contexts (e.g., exhibit shifts in the cells’ preferred firing locations). C) Proportion of feature-insensitive (FI) and feature-sensitive (FS) cells recorded on day 1 (*n* = 925), day 2 (*n* = 915 cells), and day 3 (*n* = 829). D) Examples of average aligned maps of FI (left) and FS (right) cells recorded from single-units (top) and calcium-imaging (bottom), along with the corresponding correlation values between the average aligned maps. FI cells show an average correlation of 0.654 ± 0.003 between averaged aligned maps across contexts and FS cells show an average correlation of 0.160 ± 0.006 (*M* ± SEM). E) Cumulative proportion of correlations of within context (yellow) and across context (purple) trial comparisons for FI cells (solid curves) and FS cells (dotted curves).

Our behavioral results demonstrate that animals successfully use the non-geometric features to discriminate the two contexts beginning on day 1, which is evident in the distinct digging patterns observed in each chamber during test trials. However, it is unclear how the non-geometric features are represented in the hippocampus to support this function. One possibility is that cells display geometric alignment within each context, but display location remapping across contexts (i.e., cells have distinct place fields in each chamber, but these fields rotate 180° on a trial-by-trial basis within each chamber). A second possibility is that most cells exhibit geometric alignment that is insensitive to features, displaying stability across the identically shaped contexts, but only a small group of neurons shows feature-dependent remapping across contexts. To distinguish between these possibilities, we assessed the cells’ location remapping across contexts. Place maps were first aligned by selecting the highest correlation between 0° or 180° to maximize spatial map similarity for all pairwise comparisons in each context (Figure 3A). Then, an average aligned map was computed for each context and these two average context maps were aligned relative to each other using the best match between 0° or 180°. Context similarity was assessed by calculating pixel-to-pixel cross-correlation between the average aligned maps in each context. The population distribution of context similarity displayed a strong leftward skewness (Figure 3B, n = 2669 cells; 925 cells on Day 1, 915 cells on Day 2, 829 cells on Day 3, combined single-unit and calcium imaging analysis), indicating that while most cells displayed stability across contexts, some cells fired in dissimilar locations, displaying strong remapping across contexts.

To further examine the differences in similarity, we used a context similarity cutoff value of 0.3. Cells were called “feature-sensitive” (FS) if the correlation across contexts was less than 0.3 or “feature-insensitive” (FI) if the correlation across the contexts was greater than 0.3. Notably, while this cutoff value was selected based on remapping observations previously established (Wang et al., 2015), it allowed us to investigate if cells in opposing ends of the context similarity continuum differentially contributed to feature detection. We found that cells recorded using both tetrodes and calcium imaging displayed similar percentages of FS and FI cells across days, (Figure 3C, Figure 3S). Examples of average aligned Context A and Context B maps recorded from FI and FS cells are shown in Figure 3D.

Next, we examined the map similarity of FS and FI cells within and across context. Due to the low throughput of electrophysiological techniques, we limited correlation analyses to data recorded using calcium imaging. However, behavioral and context predictions that could be conducted with few cells incorporated both single-unit and calcium imaging data. To determine map similarity, we categorized cells as FS or FI and calculated the pixel-to-pixel cross correlation between all pairwise trial comparisons of aligned maps for each cell across days (within and across context comparisons were treated identically) and averaged within a session. Pearson’s correlation values for each cell comparison were plotted in Figure 3E. For each day of testing, we performed a separate 2 × 3 repeated measures Ranked ANOVA on the correlation values, with feature sensitivity (FI or FS) as a between cells’ factor and context comparison (Within or Across) as a within cells’ factor. The interaction between feature sensitivity and contextual condition was significant for all days [Day 1: V(1) = 57.92, p< .0001; Day 2: V(1)= 39.94, p< .0001; Day 3: V(1)= 54.44, p< .0001]. Post hoc analysis indicated that FS cells were significantly more stable within than across context during all three days of testing [Day 1: V(1)=21.26, p< .0001; Day 2: V(1) =40.34, p< .0001; Day 3: V(1)= 80.33, p< .0001], demonstrating that these cells exhibited much more location remapping across-contexts than within context during all days of training. By contrast, FI cells were stable within and across context across days; showing a difference on day 1, but not on days 2 and 3 [Day 1: V(1)=45.53, p< .0001; Day 2: V(1)= 3.24, p= .4310; Day 3: V(1)= 5.76, p= .0980].

### The population of FS cells display lower context similarity across time

**Figure 4.**
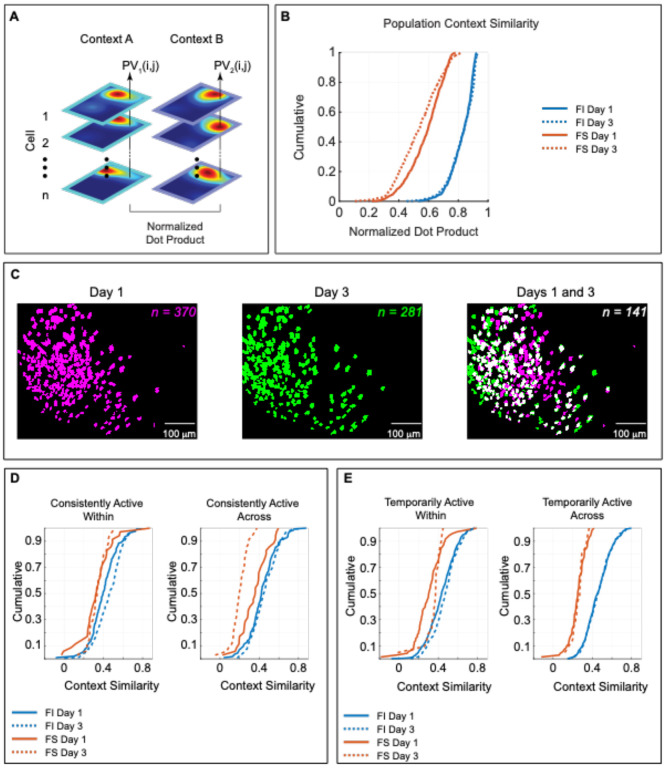
The population of FS cells display lower context similarity across time and longitudinal examination of FI and FS cell-types across days. A-B) Population activity of FS cells displays lower context similarity across time. A) Schematic illustrating dot product method. B) Distributions of population data collected from FI (blue) or FS (red) cells on day 1 (solid) or day 3 (dotted). Note higher dot products of FI cells in comparison to FS cells. FS cells have a lower dot product on day 3 than on day 1, indicating more dissimilarity in the spatial maps at the population level. C-E) Analysis of population temporal dynamics of FI and FS cells. C) Spatial footprints of cells identified from a representative animal on day 1 (magenta, left), day 3 (green, center), and cells identified on both days 1 and 3 (white, right). D) Cumulative similarity of average within (left) and across (right) trial comparisons for consistently active FI (blue) and FS (red) cells [e.g., identified on both day 1 (solid) and day 3 (dotted)]. E). Cumulative similarity of average within (left) and across (right) trial comparisons for temporarily active FI (blue) and FS (red) cells [e.g., identified only on day 1 (solid) or only on day 3 (dotted)].

The previous analysis evaluated the context similarity of single FI and FS cells for each day of training and showed that only FS displayed remapping across context, However, it is possible that representations of context at the population level may shift over time in FI cells. To determine the effect of training on population context representations, we evaluated the context similarity of FI and FS cells on day 1 vs day 3 at the population level. To this end, we calculated the normalized dot product between the Context A and Context B average aligned maps (20 × 30 cm, 600 pixels; Figure 4A) for all cells as previously described (Alme et al., 2014) and conducted two-way Ranked ANOVA with day of testing (day 1 and 3) and Feature Sensitivity (FI and FS) as between factors. Higher dot product values indicate greater similarity between population spatial representations. We found that FI population spatial representations were equally similar between contexts across days of training [Figure 4B, Day 1: n = 734, Day 3: n = 573; VT(1)= 1.21, p= .4500], whereas FS population spatial representations became more dissimilar on day 3 than day 1 [Figure 4B, Day 1: n = 100, Day 3: n = 56; VT(1)=6.1, p< .0001]. These results indicate that FS cell representations of context become more dissimilar at the population level with experience, which is important for discrimination of context.

### Longitudinal examination of FI and FS cell-types across days

Our previous analysis indicates that FS cells increase discrimination of context, whereas FI cells remain stable over time. Since studies looking at neuronal activity during memory retrieval have found that active networks are dynamic, with some cells being consistently active across time and others only being active at specific time points (DeNardo et al., 2019; Kitamura et al., 2017), we also wanted to investigate if the temporal dynamics of different cell types influenced representations of context. Calcium-imaging techniques allow tracking of the same cells across training days, making it possible to investigate the stability of neurons across time. Neurons were registered and separated according to whether they were active on both day 1 and 3, denominated *consistently active* neurons (n = 233), or active only on day 1 (*n* = 597) or day 3 (*n* = 391), denominated *temporarily active*. Figure 4C shows the spatial footprint of cells in a representative animal recorded on Day 1 (left), Day 3 (center), and the footprint overlap between days (right), corroborating the dynamic nature of active networks during reorientation. We first compared the cumulative similarity of consistently active cells (cells active on days 1 and 3) within and across context for FI and FS cells (Figure 4D), similarly to the analysis depicted in Figure 3E. Using a 3-way Ranked ANOVA with day of testing (day 1 and 3) and context comparison (within or across) as within cell factors and feature sensitivity (FI and FS) as a between cell factor, we found a significant interaction between feature sensitivity, context comparison, and day [V(1)= 19.73, p< .0001] in consistently active cells. Post hoc analyses showed that FS cells active on day 1 and 3 (*n* = 34) did not display changes within context across time [V(1)= 0.35, p= 1.0000], but they showed much higher across-context remapping on day 3 than day 1 [V(1)= 49.85, p< .0001]. This indicates that consistently active FS cells increase remapping with learning, which may facilitate feature-based context recognition. Conversely, the consistently active FI cells (n = 199) display increased stability within [V(1)= 25.44, p< .0001] and across context [V(1)= 6.85, p< .0350] over time (Figure 4D), a pattern that would facilitate consistent heading retrieval across context over time.

A similar 3-way Ranked ANOVA was conducted for temporarily active cells (cells active either on day 1 or day 3 only), but in this instance day of testing (day 1 and 3) was treated as a between factor. Results only showed a significant interaction between feature sensitivity and context comparison [V(1) = 59.33, p< .0001] and day of testing and context comparison [V(1)= 8.17, p<.0043 ]. Post hoc analyses focusing on feature sensitivity across days showed that temporary FS and FI cells were more stable within context on day 3 (FS: n = 21; FI: n= 370) than day 1 (FS: n = 70, FI: n= 527) [FS: V(1)= 2.24, p< .0499; FI: V(1)= 3.44, p< .0020; Figure 4E, left panel], but no differences were observed in FS or FI temporary cells across contexts between days 1 and 3 [FS: V(1)= 0.39, p=1.0000; FI: V(1)= 0.30, p=1.0000; Figure 4E, right panel]. These data suggest that the temporal dynamics of FS and FI also support differential roles of these cell types in context recognition and heading retrieval. Consistently active FS cells increase remapping across contexts over time, whereas temporarily active FS cells increase stability within context with training. Stabilizing representations within context, while increasing remapping across context may facilitate context recognition. Conversely, consistently or temporarily active FI cells only show increased stability within context over time, with no differences across context, an important attribute for efficient and consistent heading retrieval (e.g., heading being independent of context). These activity patterns support the idea that context recognition and heading retrieval are dissociable processes that are represented by specific cell types.

### Location remapping of FS cells is not modulated by reward location

**Figure 5.**
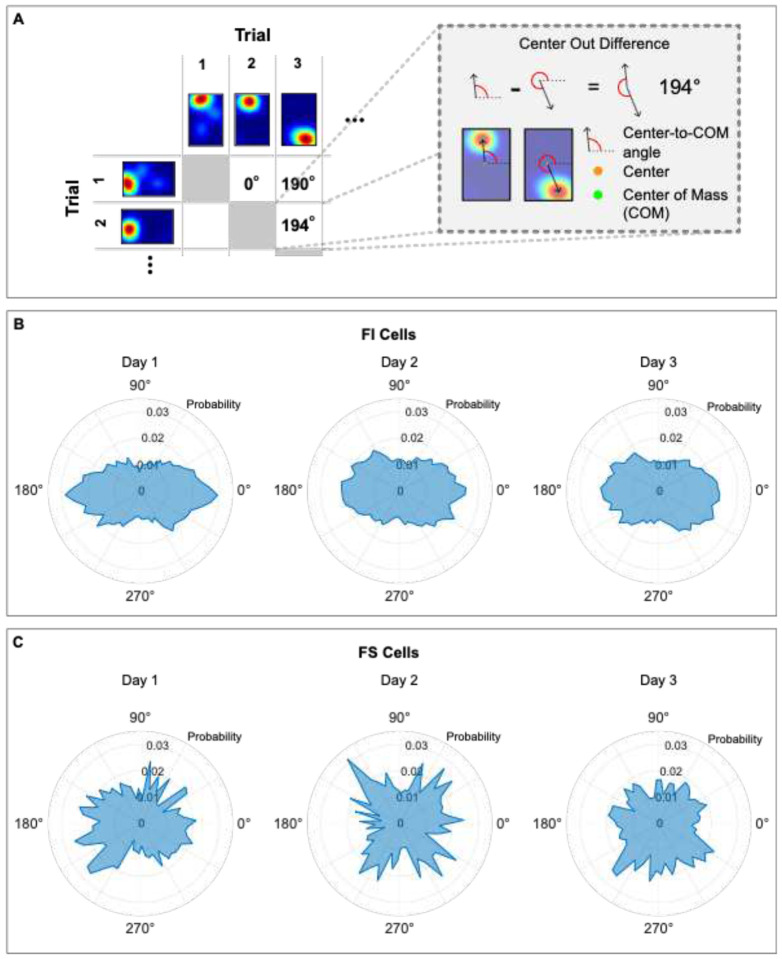
Location remapping of FS cells is not modulated by reward location. A) Schematic illustrating the center-out angle difference method. B-C) Polar histograms of center-out angle differences from pairwise comparisons generated across context for FI (B) and FS (C) cells. B) FI cells display geometric alignment indicated by peaks at 0° and 180° between the contexts on days 1 to 3. C) Conversely, FS cells do not display bimodal geometric alignment across context on any day. Although these cells display more coherent remapping across context on day 1 than on days 2 and 3, the distribution of angle differences becomes circular with progressive training, indicating that each FS cell remaps independently from each other, showing no alignment to reward location.

Having established that FS cells increase discrimination between contexts over time, but FI cells do not, we then turned to the crucial question of how FS cells shifted their preferred firing locations across the two contexts. One possibility would be that the activity of FS cells, which appeared to be sensitive to learning, might be modulated by reward location and/or respond to the reward itself (Dupret et al., 2010; Gauthier & Tank, 2018). If this were the case, the firing location of FS cells would shift coherently along distinct rewarded axes or be anchored at the position of the rewarded cup location in each context. Another possibility would be that FS cells were not modulated by reward, but rather responded to the distinct features identifying the contexts. In this case, the remapping of FS cells would show no coherence, displaying independent firing locations between contexts at the population level. To determine between these two possibilities, we calculated the angle between the center of the map and the center of mass (COM) of the place field (*center out angle*) for all trials and compared the difference between these angles for pairwise comparisons of trials across contexts (Figure 5A).

Figure 5B–C shows the distribution of center-out angle differences for pairwise comparisons of trials across contexts. As expected, FI cells rotate across context following the rotational symmetry of the rectangular chambers, resulting in bi-modal distributions with peaks at 0° and 180° across contexts (Figure 5B; Rayleigh-folded: Day 1: R(2)=405.9, p < .0001; Day 2: R(2)=366.9, p <.0001; Day 3: R(2)=361.08, p <.0001). By contrast, FS cells do not show alignment to geometry across context on any day (Figure 5C). On Day 1, the distribution of center-out angle differences departed from circularity (Rayleigh folded: Day 1: R(2)=10.10, p < .001), without showing bimodality and the observed peak did not coincide with reward location. This indicated that although these cells display some level of remapping coherence across context on day 1, this coherence is not triggered by geometry or reward location. On days 2 and 3, the angular distribution did not depart from circularity (Rayleigh folded: Day 2: R(2)=0.11, p = .90; Day 3: R(2)=0.08, p = .93), supporting the idea that the location remapping of FS cells was triggered by the distinct features and that the population of active cells was not anchored to a particular feature or location. In summary, these data indicate that FI cells display geometric alignment across contexts (i.e., coherent rotations at 0° and 180°), providing a consistent representation of geometry that would be crucial for heading retrieval. Conversely, FS cells display location remapping across contexts that is not modulated by reward, suggesting that the firing locations are tied to the different features that define each context, an attribute that is important for efficient context recognition.

### FI and FS cells display similar geometric alignment within context

**Figure 6.**
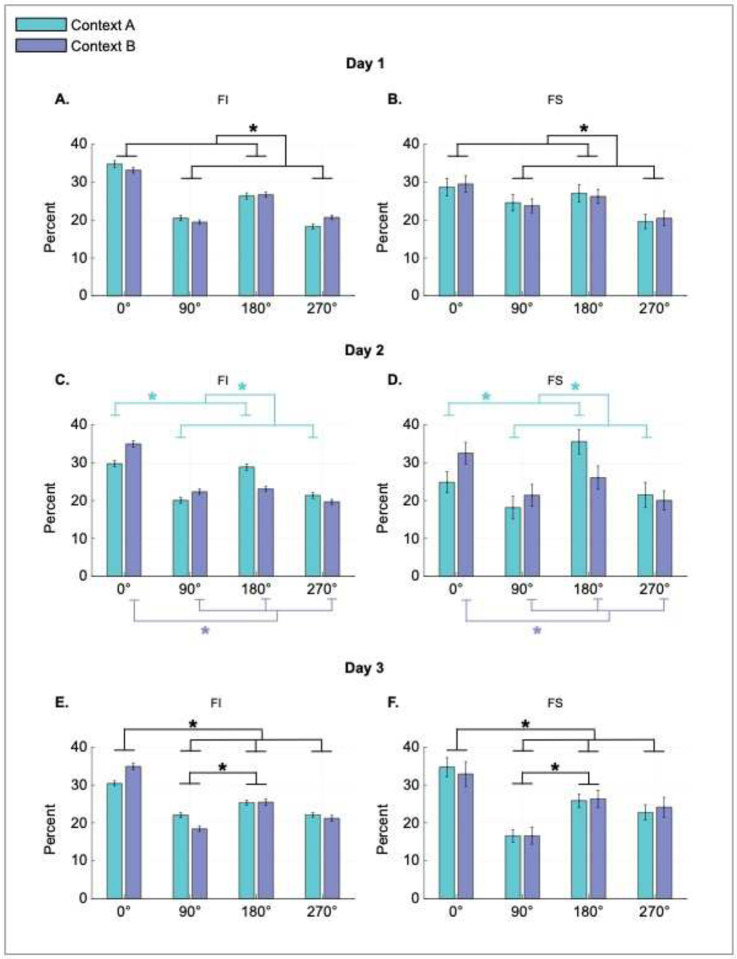
FI and FS cells display similar geometric alignment within context. A-F) Best match rotations indicate higher proportions of 0° and 180° occur more often than other rotations in Contexts A and B in FI cells (A, C, E) and FS cells (B, D, F) across days, indicating strong geometric alignment throughout training. C-D) On day 2, 0° best match rotations also occurred more often than 180° in FS cells in both contexts. On day 3, 0° best match rotations occurred more often than 180° in FI and FS cells in both contexts. Asterisks (*) represent significance using alpha = 0.05. Error bars denote ± 1 standard error of the mean (SEM).

Although the previous analyses indicated that FS cells displayed feature-sensitive remapping across context, we observed that when analyzed as a group, without distinguishing between FS and FI, the population of active cells aligned to geometry (Figure 2C). This result could happen if both FS and FI cell align to geometry within context despite having different phenotypes across context. To investigate this possibility, we evaluated the alignment of FS and FI within each context by conducting the best match rotation analysis depicted in Figure 2A, but this time broken down by chamber. This analysis was performed on the calcium imaging data to have sufficient data points for FS cells. A 4-way repeated measures ANOVA with Context (A or B) and Rotation (0°, 90°, 180°, 270°) as within cells’ factors, and cell type (FI or FS) and day (days 1–3) as between factors showed both FI and FS cells displayed geometric alignment within each context (Figure 6A–F). There was a significant main effect of rotation [F(2.89, 5827.38) = 78.27, p < .001. Additionally, there was a significant interaction between Day, Context and Rotation [F(5.83, 5884.64) = 2.86, p < .001; the complete results of this ANOVA are shown in Table S3]. In agreement with our hypothesis, post hoc Rom’s tests showed that the best match rotations at 0° and 180° occurred more often than other rotations in both Context A and Context B (p< .05 on days 1 and 3). Even though geometric alignment persisted throughout training, on day 3 both FS and FI cells also showed higher proportion of best match rotations at 0° than 180° in both contexts (p< .05), paralleling the increase in number of correct digs (see Figure 6 for multiple comparisons details). The persistent geometric alignment in FS and FI cells was also corroborated by calculating the angular difference between the center of mass of each map relative to the center of the chamber for all pairwise comparisons within context. (Figure S4). Together, our data indicate that both FI and FS cells align to geometry within context, but only FS cell display feature-sensitive remapping across context.

### FI cells predict heading, whereas FS cells predict context throughout testing

We next sought to evaluate the predictive power FI and FS cells during reorientation. We hypothesized that FI cells’ consistent alignment to geometry within and across context might serve to recover heading direction, independent of context. Conversely, the FS location remapping across context might serve to underlie context recognition.

**Figure 7.**
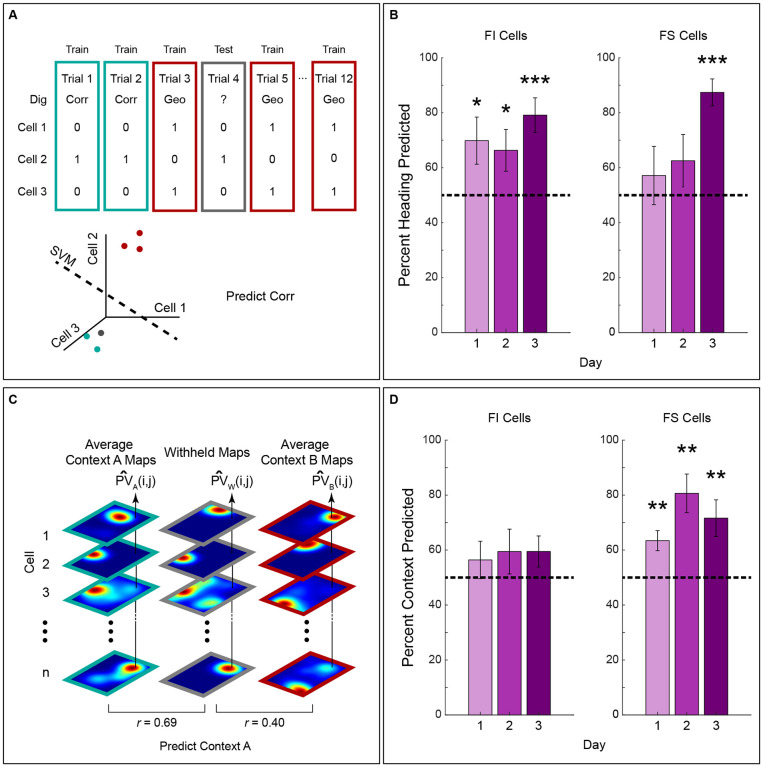
FI cells predict heading, whereas FS cells predict context. A) Schematic of heading prediction method using map orientation as predictors for each cell. A SVM classifier is trained with the binary sequence of rotations obtained through the alignment procedure shown in Figure 3 and validated using a one-trial-out procedure. B) Average heading prediction accuracy per animal across days using FI (left panel, Day 1: N = 12 mice; Day 2: N = 11 mice; Day 3: N = 10 mice) or FS (right panel, Day 1: N = 9 mice; Day 2: N = 7 mice; Day 3: N = 7 mice) cells. FI cells predict heading across time, whereas FI predict heading only on day 3, after animals learned feature/reward associations. C) Schematic of population vector analysis for predicting context. Average aligned maps in Context A and Context B are calculated withholding one trial at a time, and the dot product is calculated between normalized population vectors in each corresponding pixel across cells. The predicted context corresponds to the highest average dot product between the withheld trial and the average aligned context maps. D) Average context prediction accuracy using FI (left panel, day 1: N = 12 mice, day 2: N = 11 mice, and day 3: N = 11 mice) or FS (right panel, day 1: N = 9 mice, day 2: N = 7 mice, and day 3: N = 8 mice) cells. Only FS cells reliably predict context across days. Asterisks (*) represent significance using alpha: .05 to .001. Error bars denote ± 1 standard error of the mean (SEM).

To assess if the orientation of the place fields in FI or FS cells can be used to predict heading, we used the rotation sequence that maximized alignment of the maps (Figure 7A). This rotation sequence was determined by the series of 0° or 180° rotations that produced the maximal similarity between all trial maps for a given cell (maps were aligned within and across context). Rotation sequences were binary and indicated if a map was rotated 180° (1) or 0° (0, no rotation). The rotation sequence for C trials and G trials for all cells were used to train a support vector machine for binary classification and validated using a leave-one-out procedure. In other words, each cell’s rotation sequence was used to predict if the withheld trial corresponded to a C or G dig using the linear classifier. Prediction was assessed per trial and averaged for each session. Results indicate that FI cells predicted heading across all training days [Figure 7B, left, Day 1: t(11) =2.313, p= 0.0205, Day 2: t(10) = 2.148, p = 0.0286, Day 3: t(9) = 4.633, p < 0.0001], whereas FS cells only predicted heading on day 3 [Figure 7B, right, Day 1: t(8) =0.673, p= 0.260, Day 2: t(6) = 1.315, p = 0.118, Day 3: t(6) = 7.612, p < 0.001]. The ability of FI cells to predict heading throughout training suggests that these cells underlie heading retrieval. Conversely, the heading prediction by FS cells on day 3 corresponds to increased place field stability in each context (Figure 6F) and the formation of feature/reward associations, factors that likely improve the heading prediction accuracy of these cells.

Then, we assessed if firing location can be used to predict context. To this end, we created an average aligned Context A map and an averaged aligned Context B map for each cell, withholding one trial at a time, and calculated the average normalized dot product between the withheld map and the average aligned maps, as previously described (Figure 4A, Keinath et al., 2017). The predicted context of the withheld trial corresponded to the dot product comparison with the higher average. As expected, given the lack of remapping in the FI cells, these neurons failed to predict context above chance on any day of testing (Figure 7D, left, Day 1 to 3 = p> .05). However, FS cells predicted context on days 1 to 3 [Figure 7D, right, Day 1: t(8) = 3.668, p= 0.0032, Day 2: t(6) = 4.357, p = 0.0024, Day 3: t(7) = 3.247, p = 0.0071], providing support for the idea that the remapping of these cells is triggered by the distinct features that identify the chambers. These results suggest that FS cells serve to underlie context recognition, whereas FI cells do not. In summary, our findings show that the hippocampus contains cells with unique feature sensitivity properties that differentially predict heading retrieval and context recognition. The map orientation of FI cells, which responds to geometry independent of context, predicts heading throughout training. Conversely, FS cells, which respond to features (at least in identically shaped contexts defined by such features), predict context across days, and only predict heading once animals form feature/reward associations and these cells display stability within each chamber.

### Firing rate changes predict context and reorientation behavior by integrating featural and geometric information

Our previous analysis suggests that information about heading retrieval and context identity is partially segregated in distinct cell types, which either spatially remap in response to context-distinguishing features (FS Cells) or do not spatially remap (FI cells). Previous studies have found that trial-dependent features are often encoded through rate remapping (Allen et al., 2012). For example, a recent study examined responses in rat area CA1 after animals were exposed to a rich virtual spatial environment where levels of fog were manipulated, making the appearance of the context look different across trials. This study demonstrated that while a small subpopulation of CA1 cells discriminated contexts with or without fog by shifting the cells’ preferred firing location, similarly to the context effects we observe in FS cells, most neurons did not alter their firing location but responded to distinct levels of fog through rate remapping (Shin et al., 2022). Based on these previous results, we hypothesized that information about features and geometry could be integrated in the active network, including both FS and FI cells, through rate changes.

**Figure 8.**
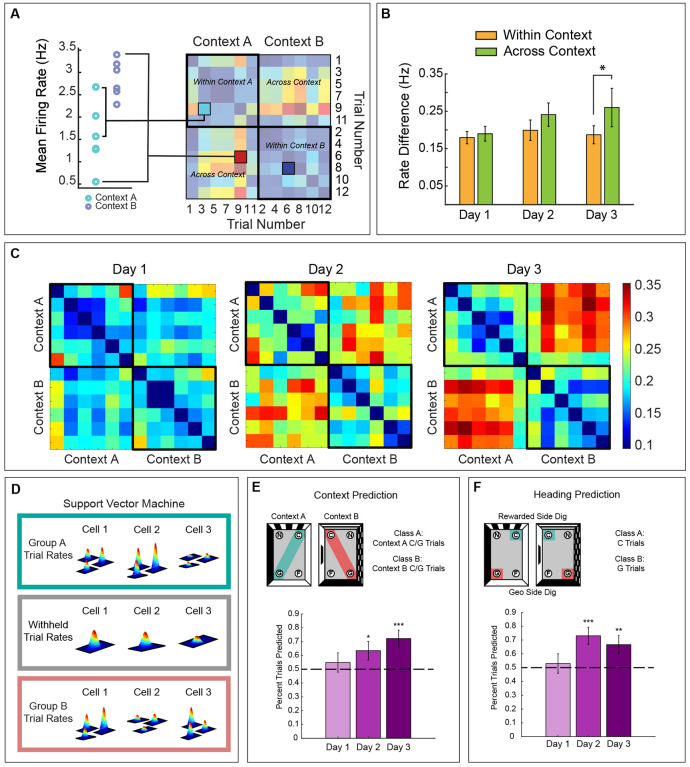
Firing rate changes predict context and reorientation behavior by integrating featural and geometric information. A) Schematic of method used to calculate rate matrices based on the absolute rate difference between trials. B) Histograms showing average rate difference within and across context trial comparisons on days 1 to 3. C) Average rate matrices across cells recorded on day 1 (left), 2 (center), and 3 (right). D) Schematic of prediction method. The mean firing rates in each trial group are used to train a support vector machine for binary classification, cross validated using a leave-one-out procedure. E) Context prediction. C or G search trials in Context A (green) and Context B (red) are used to train a SVM to predict context in a withheld trial (top schematic). Prediction accuracy across days display reliable prediction of context on days 2 and 3 (bottom histograms). F) Heading prediction. C trials and G trials in both contexts are used to predict digging side (top schematic). C and G search digs are reliably predicted on days 2 and 3, with a trend on day 1. C= correct, G= geometric error. Asterisks (*) represent significance using alpha < 0.05 or lower. Error bars denote ± 1 SEM.

To test this hypothesis, we used the mean firing rate from our electrophysiological group. We first examined within- and across-context differences in the cells’ firing rate over time to determine if changes in rate remapping mirrored the increased use of features for heading retrieval. The absolute difference in mean firing rate between trials was calculated for all pairwise comparisons for each cell and plotted as a rate matrix (Figure 8A). The across-context rate differences increased over days, but the within-context rate differences did not (Figure 8B and 8C). To quantify this effect, the average rate differences for within- and across-context comparisons were calculated for each cell. A two way repeated measures ANOVA using context comparison (within vs. across) as a within factor, day of testing (1, 2, and 3) as a between factor, and absolute rate difference per cell (rate remapping) as a dependent measure showed that there was no effect of day [F(2, 226)= 1.06, p= .3480], but a significant effect of context comparison [F(1, 226)= 10.72, p= .0010] and an interaction between day of testing and context comparison [F(2, 226)= 3.15, p= .0450]. Post hoc Rom’s tests showed that within and across comparisons were not different on day 1 (p> .05), showed a trend on day 2 (p=.07), and were significantly different on day 3 (p< .05). These data indicate that the across-context rate remapping increases with training.

To establish the relationship between the rate code and the use of features for context recognition and heading retrieval more firmly, we performed two classification analyses in our electrophysiological group. First, to examine the time course by which featural information gets incorporated into the rate code for context recognition, we tested whether firing rate on each trial predicted the context-related behavior. To do this, we identified trials in which animals correctly identified the context by digging in the correct axis (i.e., digs in C or G) and used the cells’ mean firing rate (MFR) of those trials to train a SVM for binary classification (Figure 8D). Prediction accuracy was assessed using a leave-one-out procedure for each trial and averaged for each day of training (Day 1: 51 trials, Day 2: 52 trials, Day 3: 54 trials). A one-tailed t-test revealed that firing rate did not accurately predict context on day 1, but the prediction was significant on days 2 and 3 [Figure 8E, Day 1: t(50) = 0.697, p = .245; Day 2: t(51) = 2.00, p < .0256; Day 3: t(53) = 3.612, p < .001].

To establish the relationship between the rate code and heading retrieval, we used a similar SVM classifier to determine if MFR could predict when animals dug in the rewarded vs. unrewarded side of the chamber. In this case, the MFR of all trials were classified based on behavior (rewarded side; C digs vs. unrewarded side; G digs) and assessed using a leave-one-out procedure (Figure 8F). One-tailed t-tests indicated that side prediction showed a trend on day 1 but was significant on days 2 and 3 of training [Day 1: t(50) = 0.417, p = .34; Day 2: t(51) = 3.715, p < .0003; Day 3: t(53) = 2.574, p < .0064]. These results indicate that the rate code incorporates featural information for context recognition and heading retrieval on days 2 and 3, supporting the idea that rate remapping integrates featural and geometric information for efficient reorientation.

## Discussion

In this study, we evaluated the strategies and neural representations associated with heading retrieval and context recognition using a two-context reorientation task that required attention to features to disambiguate between contexts. In agreement with our previous behavioral findings (Julian et al., 2015), we report that mice initially use geometry to retrieve heading direction and features to recognize context; however, as animals associate featural information with reward location in each chamber, they use this information to disambiguate between symmetrical locations and maximize reward. At the cellular level, we demonstrate that cells with distinct feature sensitivity differentially respond to geometric and featural cues. FI cells rapidly and persistently align to the rectangular shape of the layout, without displaying location remapping across contexts. This alignment serves to predict the side of the chamber that the animals dig for reward on a trial-by-trial basis, suggesting that these cells provide a substrate for heading retrieval. Conversely, FS cells display feature-sensitive location remapping across contexts, and this remapping serves to predict context on a trial-by-trial basis, suggesting that these cells underlie context recognition. Importantly, when FS cells display increased anchoring at 0° (i.e., increased stability) on day 3, their heading prediction accuracy increases. These data indicate that while FS cells immediately respond to featural differences through location remapping across contexts, their stability within context increases as animals make feature/reward associations. Finally, gradual changes in firing rate in the active network serve to identify context and dig location, indicating that rate remapping binds geometric and featural information. Together these data suggest that integration about heading and context involves adaptive network changes for efficient reorientation.

Our behavioral data corroborate our previous findings by showing that in relatively unfamiliar contexts, heading retrieval and context recognition rely on geometry and features, respectively (Julian et al., 2015). However, as animals learn to associate features with reward locations, these cues are used along with geometry to recover heading and localize reward within each context. The effect of experience or context size on the use of featural information to recover heading during reorientation has been well-documented (Cheng, 1986; Gouteux et al., 2001; Lee et al., 2015; Sovrano et al., 2003; Learmonth et al., 2002; Learmonth et al., 2001). However, despite the prevalence of this phenomenon, the way features are incorporated into reorientation strategies remains debated. Some researchers support a two-process modular theory, wherein reorientation is driven by a purely geometry-based mechanism, and a second process involving feature/reward associations allows navigators to disambiguate symmetrical locations (Wang & Spelke, 2002). Conversely, others have proposed that disoriented animals rely on one reorientation mechanism that can incorporate geometric or featural cues depending on their salience or predictive value (Cheng & Newcombe, 2005; Learmonth et al., 2002). Our neural data provide strong support for geometry-based reorientation, along with a second process that gradually emerges to incorporate feature/reward associations through rate remapping. However, we also find that the representation of geometry is not modular, as featural information is integrated in reorientations strategies when animals learn their directional value, which gradually modifies the alignment and firing rate of the hippocampal cells.

Feature control of hippocampal place cells recorded from disoriented animals has been extensively debated. Although many studies looked at the effects of cue cards on oriented animals (Jeffery & O’Keefe, 1999; Sharp et al., 1990), few studies completely disrupted the internal sense of direction before assessing cue card control of place fields. The first study in disoriented rats studying the effect of a cue card reported that that place cells were unstable from trial to trial, which led to the conclusion that a feature did not control hippocampal activity during reorientation (Knierim et al., 1995). Dudchenko et al. (1997) later found contradictory results showing that place fields from disoriented rats indeed followed rotations of a cue card. However, the animals in the later study were extensively trained to use features in several spatial tasks under oriented conditions, a factor that likely contributed to the subsequent use of these cues when animals were disoriented. Furthermore, since both studies tested reorientation in cylinders with minimal geometric cues, these experiments could not demonstrate the influence of geometry on hippocampal cells. Using environments with distinct shapes and rotational symmetries in the presence of non-geometric features, we previously showed that the hippocampal map aligns to geometry (Keinath et al., 2017). In the current study, we find that the geometric alignment of the hippocampal map persists even after animals increase the number of correct responses, suggesting that mice learn to combine geometry and features to dissociate geometrically equivalent locations and maximize reward.

The perception of geometry involves incidental learning independent of reinforcement, whereas gradual formation of feature/location associations requires associative reinforcement learning (Doeller & Burgess, 2008). Our neural data illustrate these learning distinctions. Starting on day 1, we observe geometric alignment of FI cells, whereas rate remapping across contexts emerges more gradually as animals learn feature/reward associations. Additionally, we also observe that FS cells shift their preferred firing location in response to context-specific featural information. This indicates that in situations of contextual ambiguity where features are the only cues that distinguish the contexts, these cues can be used in two different ways during reorientation: 1) Context recognition, a process that does not require associative learning. 2) Formation of feature/location associations, a process that requires reinforcement learning and facilitates heading retrieval.

Our analysis of stability across context reveals that FI cells are stable, whereas FS cells are not. It has been previously suggested that place cell stability is a correlate of memory because the ability of a cell to fire in the same location over time requires some of the same molecular cascades and modulatory factors as memory consolidation (Agnihotri et al., 2004; Kentros et al., 2004; Muzzio et al., 2009). However, a recent study looking at cells expressing cFos, a marker of cellular activity associated with memory formation (Kitamura et al., 2017), showed that the hippocampus contains subpopulations of cells with distinct stability patterns that differentially express this early gene (Pettit et al., 2022; Tanaka et al., 2018). The authors suggested that place cell remapping provides an index of memory that changes with task contingencies and situations, whereas stability codes aspects of an event or context that are more static in nature (Tanaka & McHugh, 2018). This interpretation supports our current findings showing that FS cells shift their preferred firing location in response to the distinct features and task-contingencies (e.g., remapping across context, but becoming more stable within context as animals learn feature/location associations). Conversely, FI cells likely respond to the stable shape of layout and thus contribute to heading retrieval.

Analysis of the same neurons over time using calcium traces showed that FI cells show the same stable pattern within and across contexts regardless of whether they are consistently active (i.e., present at the beginning and the end of training) or temporarily active (i.e., only present on specific days of training). However, FS cells respond in different ways depending on their activity pattern. The consistently active FS cells increase location remapping across contexts at the single cell and population level as animals gain experience with the task, a phenomenon that likely serves to distinguish the contexts. Conversely, the temporarily active FS cells respond in a context-specific manner by showing increased stability within context over time, a phenomenon that likely reflects the formation of feature/reward associations within each context. These findings suggest that FS cells respond to features for both context recognition and feature/reward associations through distinct activity patterns (i.e., consistently, or temporarily active).

Since information about geometry and features appears to be segregated in cells with distinct sensitivity characteristics, a neural mechanism must integrate this information for efficient heading retrieval. Rate changes in pyramidal cells have been shown to code trial-dependent features. Rats tested in a T maze, where the identity of food reward guided behavior, displayed changes in firing rate in response to the type of food reward or previous location of the animal in the maze, without alterations in spatial selectivity (Allen et al., 2012). Rate changes occurring in the absence of location remapping have also been reported when a recording chamber was varied, by alterations in shape (circular versus square) or wall color (black versus white), but its spatial location remained unchanged (Leutgeb et al., 2005). These results indicate that rate remapping serves to code task contingencies and/or featural information within global representations of context. Here, we observe that changes in firing rate across contexts increase as animals learn to incorporate feature/reward associations within each context. Importantly, these changes occur in all cells, including those that only align to geometry, suggesting that rate remapping is the mechanism that serves to bind task-relevant cues.

Featural and geometric information may be conveyed to the hippocampus through different input streams. The postsubiculum is involved in landmark control of head direction cells, suggesting that this area provides information about the directional value of features (Yoder & Taube, 2011). Additionally, fMRI studies in humans indicate that the right dorsal striatum is involved in coding locations of landmarks (Doeller et al., 2008). However, other regions, such as the retrosplenial cortex and parahippocampal place area play important roles in representing distinct aspects of scenes and landmarks (Epstein, 2008; Epstein & Baker, 2019; Epstein et al., 2017; Julian, Keinath, Marchette, et al., 2018; Persichetti & Dilks, 2019) and boundary information is found in retrosplenial cortex, medial entorhinal cortex, postrhinal cortex, and parasubiculum (Alexander et al., 2020; Gofman et al., 2019; Julian, Keinath, Frazzetta, et al., 2018; Savelli et al., 2008; Solstad et al., 2008). Segregation of featural/landmark and geometric information in different brain regions was also found in an fMRI study using an adapted reorientation task (Sutton et al., 2010). Here, we observe that information about geometry and features is integrated in area CA1 and that feature-sensitivity in distinct CA1 cell groups differentially contributes to context recognition and heading retrieval. Our data is in agreement with recent findings showing that CA1 subpopulations with distinct remapping properties differentially participate in context recognition vs. perception of cue alterations (Shin et al., 2022), suggesting that CA1 is the ideal substrate to integrate cognitive processes for efficient performance.

In summary, this study provides important information about the neural architecture underlying reorientation and the temporal dynamics and characteristics of the neural processes underlying heading retrieval and context recognition.

## Star Methods

### Subjects –

Male mice (C57BL/6J, Jackson Laboratory, Bar Harbor, ME) were placed on a 12-hour light/dark cycle and all experiments were carried out during the light portion of the light/dark cycle Animals were food deprived until reaching 85% of their ad libitum body weight and food deprivation continued until the last day of testing. Animal living conditions were consistent with the standard required by the Association of Assessment and Accreditation of Laboratory Animal care. All experiments were approved by the Institution of Animal Care and Use Committee of the University of Texas at San Antonio.

### Two-Context reorientation task –

Training began with a shaping phase during which food restricted mice were exposed to the reward buried in odor-masked bedding in a medicine cup placed inside their home cages at least 4 days prior to the commencement of the experiments. Mice received 12 trials per day that alternated between the two contexts. On the first day, the reward was placed on top of the bedding in the rewarded corner for the first 4 trials (2 trials in each context) and was then buried at sequentially deeper positions for the next 4 trials to train the animals to discriminate the contexts and associate each context with a distinct reward location (2 in each context). Animals were tested on the last 4 trials of day 1 and the 2 following days (12 trials per day). During testing trials, the reward was buried 1.5 cm from the surface of the cup, and animals received equal number of trials in each context. Before each trial, each mouse was placed in a cylinder with a removable base that was placed on a turntable platform. To disorient the animal, the table was rotated 4 clockwise and 4 counterclockwise revolutions. The animal was then transported to the reorientation context, and the base was removed from underneath the animal, which allowed animals to enter the chamber facing random directions and marked the commencement of the trial. In all trials, animals remained in the chamber until they found the reward. The chambers were rotated 90° after each trial to prevent the use of any extraexperimental cue. During the inter-trial interval (3–5 min) the chambers were thoroughly cleaned with ethanol to remove odor trails and all the cups were refilled with clean scented bedding. The location of the first dig was used as the dependent measure for reorientation. First digs occurred in one of the four corners of each chamber: The correct corner (C) containing the reward, the near corner (N) adjacent to the same short wall as the rewarded corner, the geometric corner (G) that was the rotational equivalent corner as the rewarded corner, and the far corner (F) adjacent to the same long wall as the rewarded corner (Figure 1A). Digs were counted when an animal displaced bedding using both front paws. Digs were scored by two experimenters blind to the experimental condition, and in the event of a dispute, a third experimenter confirmed the dig location.

### Surgeries –

Mice were anesthetized with isoflurane (3%; 1L/min O_2_ flow) and maintained at 1.5% isoflurane for the duration of surgery. Mice were placed in a stereotaxic frame (David Kopf Instruments) in a flat skull orientation. Body temperature was regulated using a heating pad and eyes were kept moist using a lubricant (Puralube Vet Ointment). Mice were administered 5 mg/kg Rimadyl pre-operatively and for two days following surgery.

#### Electrophysiology surgery:

Mice were implanted with 6-tetrode microdrives above the right dorsal hippocampus (from bregma, AP: −1.6 – 2.1, ML: 1.6, DV: −1.0, from pia). A ground wire was connected to a screw placed in the contralateral occipital plate. The microdrives were affixed to the skulls with cyanoacrylate and dental cement.

#### Calcium imaging surgery:

Mice were transfected with 400 nl of the calcium indicator GCamp6f (AAV1-hSyn-GCamp6f-WPRSE.SV40, Addgene; titer: ≥ 1×1013 vg/mL) in dorsal hippocampal subregion CA1 (from bregma, AP: −2.1, ML: 1.8, DV: −1.65) at a rate of 50 nl / min using a Hamilton Syringe (CAT: 86257). A 2 mm diameter craniotomy was centered over AP: −1.9, ML: 1.7. Superficial cortical tissue above CA1 was gently aspirated via sterile cold saline irrigation and a 1.8 mm diameter gradient refractive index (GRIN) lens (Edmund Optics, 64–519) was implanted over CA1. The lens was lowered to a depth of −1.20 mm from the surface of the skull and depressed an additional 50 μm to compensate for any brain swelling during surgery. A stainless-steel screw was placed over the contralateral occipital plate and cyanoacrylate and dental cement were used to stabilize the GRIN lens. A surgical silicone adhesive (Kwik-Sil, World Precision Instruments) was applied to the exposed GRIN lens for protection as animals recovered from surgery. At least 4 weeks post-injection, animals underwent a base-plating procedure in which an aluminum baseplate was affixed to the skull. During base-plating, a miniaturized fluorescent endoscope (v3; miniscope.org) was attached to image calcium events in the anesthetized animal to find the optimal field of view containing visible cell structures. Baseplates were cemented to the skull to maintain the field of view using dental cement. After the baseplate was secured, a black Delrin cap was magnetically attached to the baseplate to protect the GRIN lens while the mice were not being recorded. Optimal focal points were determined the following day once the dental cement has fully cured.

#### Electrophysiological Recordings:

Two weeks after surgery, animals were placed in an environment distinct from task-related arenas, and an experimenter searched for hippocampal CA1 pyramidal cells. The microdrive was connected to a tethered unity gain amplifier with green and red LEDs for tracking the position of the animal (tracking was also extracted using DeepLabCut, see below). The units were amplified between 2500 and 10,000 and filtered between 400 and 9000 Hz. The amplifier output was digitized at 32 kHz. The position of the animal and its electrophysiological data were recorded by Cheetah Data Acquisition software (Neuralynx, Bozeman, MT). The electrode bundle was lowered by 5–15 microns per day until pyramidal cells were identified by their characteristic firing patterns (Ranck, 1973). Cluster quality and place cell waveform characteristics were checked using MClust software (developed by A. David Redish, University of Minnesota). Cells were included if they formed isolated gaussian ellipses with minimal overlap with surrounding cells and noise and waveforms were stable throughout the session. All cells were also inspected to rule out the presence of spiking events during the 2 ms refractory period. No attempt was made to track the same cells across days. Cells were isolated using action potentials recorded in all trials within a training day to minimize per-trial bias and to track cells across trials.

After all data were collected, electrode placement was verified by deeply anesthetizing the animal and passing a current (0.1 mA for 5 seconds) through the tetrodes (52500 Lesion Making Device, Ugo Basile) and perfusing the animal with ice cold 0.1 M PBS followed by 4% paraformaldehyde (PFA) made in 0.1 M PBS. The brains are placed in 4% PFA containing 3% ferrocyanide overnight, then incubated overnight in 30% sucrose-azide solution, cryosectioned (50μm, coronal) and collected on slides.

#### Calcium-Imaging recordings and analysis:

Behavioral training began once optimal focal points were determined. The miniendoscope was attached prior the commencement of each session and unattached at the end of recording, before animals were returned to the home cage. During the experiments, the tether connecting the miniendoscope was attached to commutator that allowed animals to move freely while reorienting. After behavioral experiments were concluded, animals were perfused in the same manner as the animals used for electrophysiology, and histology was confirmed by the presence of green fluorescent protein in CA1. Behavioral data were extracted using DeepLabCut (Mathis et al., 2018; Nath et al., 2019). Calcium-images in each session were concatenated across trials and motion corrected (Pnevmatikakis & Giovannucci, 2017). Individual cells were identified, denoised, and deconvoluted using a constrained nonnegative matrix factorization algorithm previously reported (Pnevmatikakis et al., 2016; Sheintuch et al., 2017; Zhou et al., 2018). Inferred likelihood of spiking events (ILSE) were obtained from these deconvolutions and used as the measure of cellular activity.

### Calcium imaging cell registration –

Cells identified on each day of recording were registered across days to allow longitudinal analysis of activity patterns across long time periods. Cells were registered using a probabilistic approach (CellReg; Matlab) (Sheintuch et al., 2017). Shape metrics of spatial footprints of registered cells (circularity, size, number of contiguous pixels) were computed and used to train a naïve Bayesian classifier against a subset of visually confirmed spatial footprints that were “cell-like”. Only cells that were classified as cell-like were included in subsequent analysis.

### Place Field Analysis –

First, animal position data was aligned so that the short wall adjacent to the rewarded corner is in the same location in each context across trials. An experimenter marked the boundaries of the chamber from position data and known trial chamber orientation, then applied a coordinate transformation (homography), converting the position data from video coordinates to physical coordinates (cm). Place fields were calculated from cell activity measured by spike times (electrophysiology) or by ILSE (calcium-imaging). The chamber was binned into 1 cm × 1 cm pixels and the number of spikes or summed ILSE that occurred in each pixel were counted to measure the activity map. The time spent in each pixel was calculated to measure the time map. The activity map is divided by the time map and then smoothed with an isometric Gaussian kernel (σ 3 cm), resulting in the final place map. Pixels sampled for at least 0.05 seconds after smoothing were considered sampled. Only data from periods of movement that exceeded 2 cm/s were included for analysis.

### Context Similarity Analysis –

Place maps from disoriented animals align to geometry, displaying 180° rotations between trials. To assess context similarity, maps are aligned to account for 180° rotations due to geometry. The maps recorded in each context were first aligned by selecting the best sequence of rotations that maximized similarity for each pairwise comparison. This was done by comparing correlations without rotation of the second map (0°) or rotating it 180°. This procedure generated a binary sequence that represented the optimal alignment in each context. Then, an average aligned map was calculated for each context. The average context maps were, in turn, aligned relative to each other by selecting the maximal correlation between 0° or 180°. If the average maps were rotated, then the binary sequence of the rotated map was adjusted accordingly (e.g., 0,1,0,0,0,1 would become 1,0,1,1,1,0). Correlations between the average aligned maps were used to classify FS and FI cells.

### Population similarity across context –

To calculate similarity of population representations of Context A and Context B, a population vector was computed by stacking each cell’s average aligned map in each context. The normalized dot product between each corresponding pixel across average aligned maps provided a measure of similarity in each population (FI vs. FS cells). High dot product indicated high similarity between average aligned maps in the population.

### Heading prediction using map orientation of FI and FS cells –

Heading was predicted using place field orientations calculated from the context similarity analysis. For each cell, the sequence of rotations that produced the maximal spatial correlation between 0° and 180° was used. Rotation sequences were binary and indicated if a trial map was rotated (1) or not (0), for example: 0,1,0,0,1,0 would indicate that on trial 2 and 5 the maps were rotated 180°. Then, the rotation of maps of C search trials and G search trials were segregated and used to train a support vector machine (SVM) for binary classification. The trial rotation for each cell was used as a predictor. The SVM was cross-validated using a leave-one-out procedure and the percent of correctly predicted trials were averaged for each session.

### Context prediction using firing location of FI and FS cells –

Context was predicted by comparing the maps in a withheld trial and the average aligned maps in Context A and Context B. The average aligned maps were calculated by averaging the location activity of each cell in each context, excluding the withheld trial, in each cell group (FI vs. FS). Next, the average normalized dot product was computed between all the cells’ maps in the withheld trial and the average aligned maps. The average aligned maps with the highest average dot product with the withheld trial maps predicted context for that trial. Correctly predicted trials were averaged for each session.

### Context prediction using firing rate –

Trials in which animals made C or G responses were separated into two classes: Context A and Context B. Then, the mean firing rates (MFR) were used to train a support vector machine (SVM) for binary classification. A linear kernel was used with an empirical prior to train the SVM, which was cross-validated using a leave-one-out procedure (e.g., having one trial withheld at a time). Correctly predicted trials were averaged for each day.

### Heading prediction using firing rate –

Heading prediction was accomplished using the same procedure as above, except that the trials were separated into two classes based on digging location. C dig trials were grouped in one class and G dig trials were grouped in the other. Using a leave-one-out cross validation procedure, the support vector machine predicted whether the animals’ digging side corresponded to the rewarded or geometrically equivalent locations. Correctly predicted trials were averaged for each day.

### Center-out polar plots –

For each cell, the series of maps in both contexts were first aligned in relation to the feature on short wall next to the reward. Then, an angle from the center of the rectangle to the center of mass of the place-field was calculated for each trial. Angle measures increased counterclockwise (CCW), beginning with 0°. The center-out angular difference between each pair of maps was calculated as the amount of CCW rotation needed to align the second map with the first. If the center-out difference included different contexts, then the second map was always context B. A probability distribution, in 6° bins, was then calculated for each data set [day of training (1–3), feature sensitivity (FI/FS), and comparison type (within/across)]. Each distribution was then represented in a polar plot. For center-out statistical analyses, an angle doubling procedure was applied to account for the bimodality of most distributions (Fisher, 1995), wherein the value of each angle was doubled. This step created unimodal distributions that could be tested to determine if they violated circularity. If the doubled angle was larger than 360°, then 360 was subtracted from this value, and if it was not, the doubled angle itself was used.

### Statistical analysis

#### a. Behavioral analyses

All data was checked for normality using the Shapiro-Wilk test and included Greenhouse-Geisser (G-G) corrections for sphericity violations. We used two-way repeated measures ANOVA for behavioral analysis of dig locations. The G-G approximation allowed us to correct the original degrees of freedom (df) by epsilon, the degree to which the sphericity was violated. For example, in the ANOVA of dig locations, we would multiply 3 and 39 (the original df) by the estimated value of epsilon at 0.7271, generating the adjusted df 2.18 and 28.36, respectively for the numerator and denominator of the corrected F value. Behavioral performance was further evaluated using Bayes Factors (BF) (Kruschke, 2015) to determine whether animals were digging at random (model M_null_), or preferentially based on learning (model M_alt_). Both models are based on Bernoulli distributions, where the likelihood function for dig outcomes for each day and mouse is defined as:

$$p(z,N|\theta )=\theta^{z}{\left(1-\theta \right)}^{N-z}$$

N represents the total number of trials, z is the number of trials with correct outcomes (digs in C), and θ is the probability of a successful outcome. Digging patterns were evaluated considering 2 digging patterns: A) Proportion of digs in the geometrically correct axis in each context digs (C/G Model); B) Proportion of digs in the correct (C) corner (C Model). We define the models as follows:

##### C/G Model:

M_null_: This model represents that the animal has not learned the task and the outcomes are the result of 50–50 chance (expressed as = 0.5):
$$p\left(M_{\text{null}}\right)=0.5^{z}{\left(1-0.5\right)}^{N-z}$$
M_alt_: This model represents that the animal has learned the task, indicated as a probability between 0.5 and 0.9. In order to achieve a uniform distribution across the range of values, then an integration over probability θ is calculated as follows:
$$p\left(M_{\text{alt}}\right)=\frac{1}{0.9-0.5}\int_{0.5}^{0.9}\theta^{z}{\left(1-\theta \right)}^{N-z}d\theta$$

The Bayes Factor for each animal (represented by ‘i’) is calculated as follows:

$$BF_{i}=\frac{p\left(M_{\text{alt}}\right)}{p\left(M_{\text{null}}\right)}=\frac{\frac{1}{0.9-0.5}\int_{0.5}^{0.9}\theta^{Z}{\left(1-\theta \right)}^{N-z}d\theta }{0.5^{Z}{\left(1-0.5\right)}^{N-z}}$$

##### C Model:

M_null_: This model represents that the animal has not learned the task and the outcomes are the result of chance (25% of the time a mouse would go to each of the 4 corners, expressed as *θ* = 0.25):
$$p\left(M_{\text{null}}\right)=0.25^{z}{\left(1-0.25\right)}^{N-z}$$
M_alt_: This model represents that the animal has learned the task, indicated as a probability between 0.25 and 0.9 (above chance):
$$p\left(M_{\text{alt}}\right)=\frac{1}{0.9-0.25}\int_{0.25}^{0.9}\theta^{z}{\left(1-\theta \right)}^{N-z}d\theta$$

We assume, a priori, that both models are equally probable, p(M_alt_)=p(M_null_), and so the BF for a given animal (represented by ‘i’) is calculated as.

$$BF_{i}=\frac{p\left(M_{\text{alt}}\right)}{p\left(M_{\text{null}}\right)}=\frac{\frac{1}{0.9-0.25}\int_{0.25}^{0.9}\theta^{z}{\left(1-\theta \right)}^{N-z}d\theta }{0.25^{Z}{\left(1-0.25\right)}^{N-z}}$$

For a group of independent subjects, in both model C and model C/G, we calculate the group’s BF as:

$$BF_{\text{group}}=BF_{1}\cdot BF_{2}\cdots BF_{N}=\prod_{i}^{N}BF_{i}$$

A conventional hypothesis decision threshold states that when log(BF) > log(3) ≈ 1.1, there is$ substantial evidence for M_alt_ (learning), and when or log(BF) < log_e_(1/3) ≈ −1.1 there is substantial evidence in favor of M_null_ (non-learning), where log is the natural logarithm (Kruschke, 2015). We plot the log(BF)s as cumulative distributions and denote the critical values as vertical dotted lines (Gallistel et al., 2014).

#### b. Neuronal analyses

Two-way repeated measures ANOVAs were used to analyze place field alignment over time of cells recorded with tetrodes or calcium imaging. Repeated measures Ranked ANOVAs were implemented to analyze the geometric alignment of FI and FS cells (Brunner et al., 2002). Ranked ANOVAs have power advantages in comparison to other tests (Wilcox, 2022), especially when experimental conditions have distributions that are sensitive to differences in the average ranks. Three-way repeated measures Ranked ANOVAs were used for consistently and temporary active FI and FS cells. Dot product population vectors were analyzed with a two way Ranked ANOVA variant described in previous work (Konietschke et al., 2015; Wilcox, 2022). All analyses involving FI and FS cells included Type III sums of squares corrections for unbalanced factorial ANOVAs, given the uneven sample size of the subpopulations. Rom’s post-hoc tests were used for all regular ANOVAS and multiple comparisons with Bonferroni corrections for ranked tests (Noguchi et al., 2012). Single sample t-test were used for support vector machine classification. Rate remapping was analyzed using a two-way repeated measures ANOVA. Distributions of center-out doubled angles were analyzed using the Rayleigh test. ANOVA statistical analyses were performed in the R programming environment (R. Core Team, 2022). Statistical decisions were made using a significance probability set at 0.05.

## Supplementary Material

1
