# Supplementary material for: Distinct neural mechanisms for heading retrieval and context recognition in the hippocampus during spatial reorientation
Source: Res Sq. 2023 Mar 31:rs.3.rs-2724785. Preprint. [Version 1] doi: 10.21203/rs.3.rs-2724785/v1 (PMC10081367; doi:10.21203/rs.3.rs-2724785/v1)
Supplement: 1 [file NIHPPRS2724785V1-supplement-1.pdf]

Table S1

Condition	Subject	Log (BF)		Condition	Subject	Log (BF)		Condition	Subject	Log (BF)
Day 1	AK42	0.2587		Day 2	AK42	0.0529		Day 3	AK42	-1.3172
	AK74	-1.4664			AK74	1.0563			AK74	-0.7228
	CMG87	0.2587			CMG87	-0.3758			CMG87	-0.7228
	CMG89	-0.7739			CMG89	2.9944			CMG89	-0.4658
	CMG129	-0.7739			CMG129	0.6299			CMG129	-0.7228
	CMG154	-0.4009			CMG154	1.1714			CMG154	-1.4844
	CMG159	-0.7739			CMG159	1.5516			CMG159	0.0529
	CMG161	0.2587			CMG161	-0.3758			CMG161	1.0563
	CMG162	-0.7739			CMG162	0.0529			CMG162	3.8949
	CMG169	1.7795			CMG169	1.8580			CMG169	2.9944
	HG1	1.7795			HG1	0.4350			HG1	5.8906
	JJ9	-1.4664			JJ9	1.0563			JJ9	3.8949
	KL1	-0.7739			KL1	1.0563			KL1	2.9944
	MG1	0.2587			MG1	3.8949			MG1	2.3272
	Global	**-2.6096**			Global	**15.0582**			Global	**17.6698**

Table S1 (complement of Figure 1E). Individual and global Bayes Factors (BF) to test the alternative model (M_{Alt}) that animals dug in the correct cup location vs. the null model (M_{null}) that animals dug by chance [log(BF)> 1.1 provides credibility for $M_{Alt,,}$ log(BF)< -1.1 provides credibility for M_{null}].

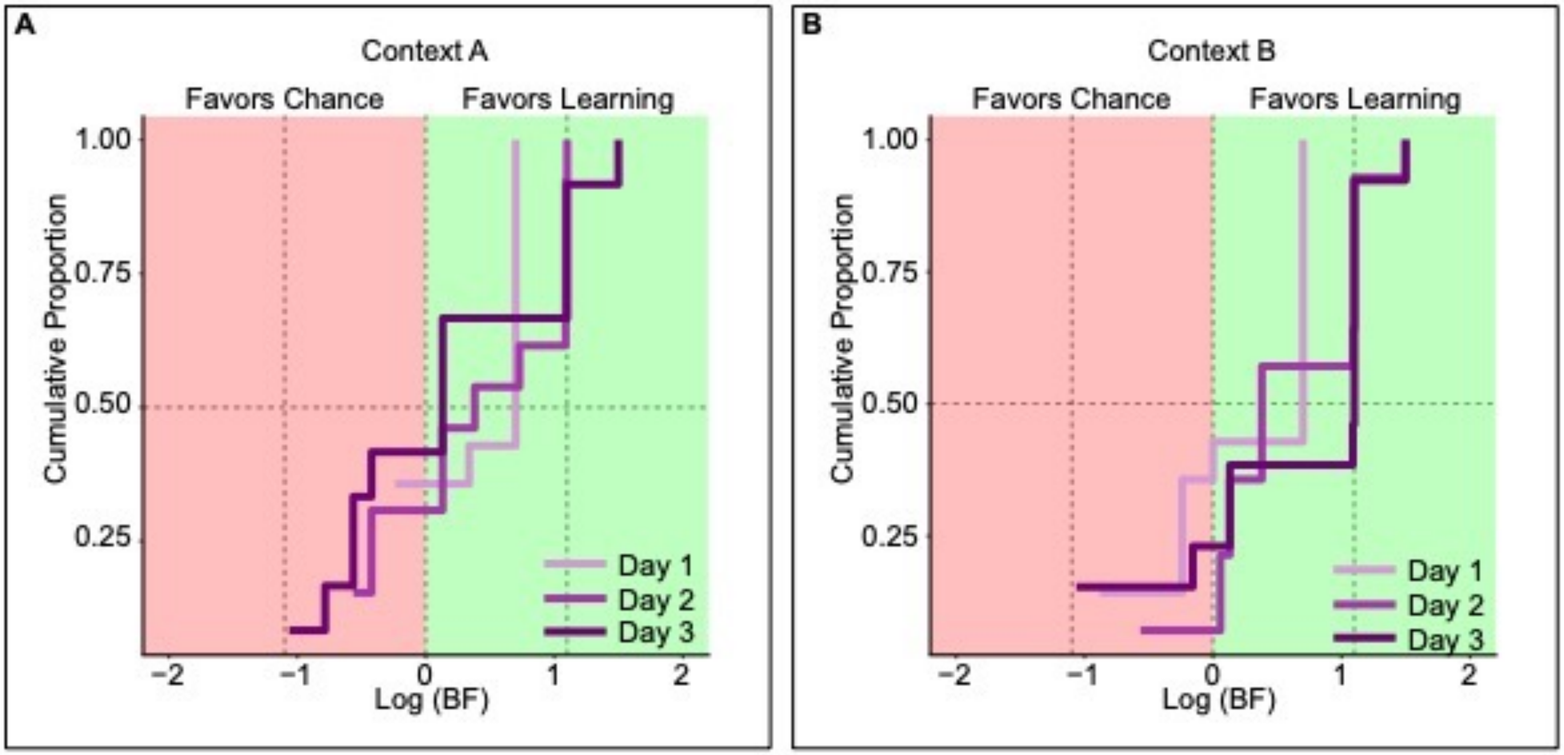

Figure S1. Cumulative proportion of Bayes Factor (BF) on days 1 to 3 per context. The BFs evaluated the alternative model (M_{Alt}) that animals preferentially dug on the rewarded axis in each context (long wall right in Context A and long wall left in Context B, Figure1) vs. the null model (Mnull) that animals dug by chance. The cumulative function across subjects in each context as well as the global BF analysis showed similar credibility in support of M_{Alt} in both contexts across days [Context A: group BFs (days 1, 2, and 3): log (BF)= 4.16, 7.23 and 6.94; Context B: group BFs (days 1, 2, and 3): 4.18, 8.60, and 8.56; for details on individual data see Table S2]. These results indicate that BF associated with the long wall right axis has credibility over the long wall left axis in Context A, but the opposite happens in Context). Conventional values showing the border marking credibility for the M_{null} (chance digging) and the M_{alt} (use of geometry) are indicated by vertical dashed lines. The value of half of the sample (0.5) is marked by a horizontal dashed line. Log BF(3) > 1.1 indicate credibility for M_{alt} (geometric learning), whereas log BF(1/3) < –1.1 indicate credibility for M_{null} (chance performance).

Table S2

Condition	Subject	Log (BF)
Context A Day 1	AK42	0.6998
	AK74	-0.2400
	CMG87	0.6998
	CMG89	-0.2400
	CMG129	0.6998
	CMG154	0.6998
	CMG159	-0.2400
	CMG161	0.6998
	CMG162	-0.2400
	CMG169	-0.2400
	HG1	0.6998
	JJ9	-0.2400
	KL1	0.6998
	MG1	0.6998
	Global	**4.1586**

Condition	Subject	Log (BF)
Context A Day 2	AK42	0.1305
	AK74	-0.5651
	CMG87	0.3799
	CMG89	1.0990
	CMG129	-0.4227
	CMG154	1.0879
	CMG159	0.7277
	CMG161	-0.4227
	CMG162	0.1305
	CMG169	1.0879
	HG1	1.0879
	JJ9	-0.5651
	KL1	1.0990
	MG1	2.3753
	Global	**7.2299**

Condition	Subject	Log (BF)
Context A Day 3	AK42	0.1305
	AK74	2.3753
	CMG87	-0.5651
	CMG89	-0.1580
	CMG129	0.1305
	CMG154	-0.4227
	CMG159	0.1305
	CMG161	-1.0643
	CMG162	1.0990
	CMG169	-0.7839
	HG1	1.4983
	JJ9	1.0990
	KL1	1.0990
	MG1	2.3753
	Global	**6.9434**

Condition	Subject	Log (BF)
Context B Day 1	AK42	0.6998
	AK74	0.6998
	CMG87	0.6998
	CMG89	0.6998
	CMG129	0.6998
	CMG154	-0.5108
	CMG159	0.6998
	CMG161	-0.2400
	CMG162	-0.2400
	CMG169	0.6998
	HG1	0.6998
	JJ9	-0.8835
	KL1	-0.2400
	MG1	0.6998
	Global	**4.1839**

Condition	Subject	Log (BF)
Context B Day 2	AK42	-0.5651
	AK74	1.0990
	CMG87	0.3799
	CMG89	0.7277
	CMG129	0.3799
	CMG154	1.4983
	CMG159	0.1305
	CMG161	0.3799
	CMG162	0.1305
	CMG169	1.0879
	HG1	0.0570
	JJ9	1.0990
	KL1	1.0990
	MG1	1.0990
	Global	**8.6024**

Condition	Subject	Log (BF)
Context B Day 3	AK42	0.1305
	AK74	-1.0643
	CMG87	1.0990
	CMG89	0.1305
	CMG129	-1.0643
	CMG154	1.0879
	CMG159	0.1305
	CMG161	1.0990
	CMG162	1.0990
	CMG169	1.0990
	HG1	1.4983
	JJ9	2.3753
	KL1	-0.1580
	MG1	1.0990
	Global	**8.5614**

Table S2 (complement of Figure S1). Individual and global Bayes Factors (BF) to test the alternative model (M_{Alt}) that animals preferentially dug on the correct axis in each context (long wall right in context A and long wall left in context B, Fig.1) vs. the null model (M_{null}) that animals dug by chance.

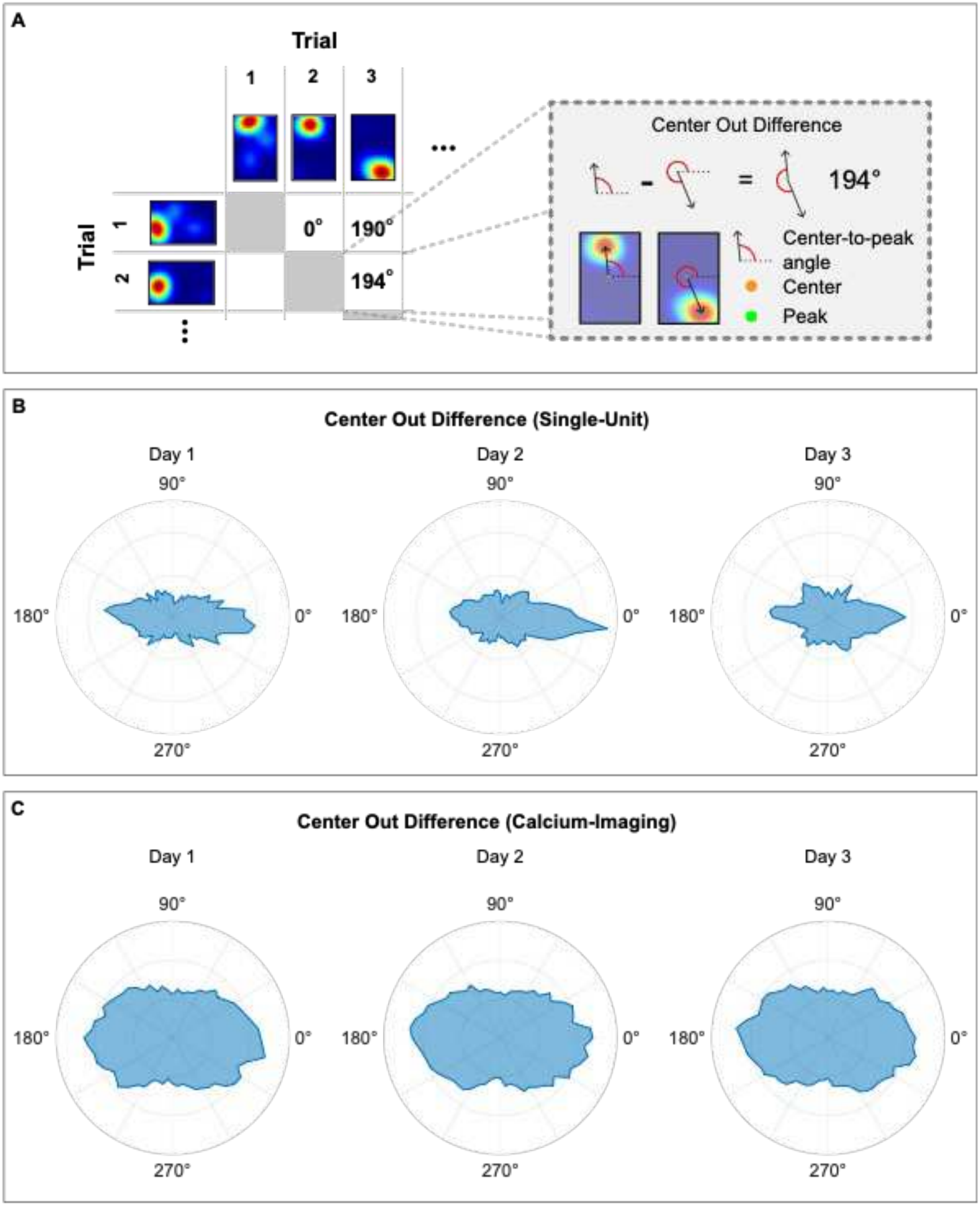

Figure S2. Place field alignment to spatial geometry persists over days. A) Schematic of center-to-peak angle analysis. For each pairwise comparison, the angle from the center of the map to the center of mass (centroid of the field) is calculated from both maps, and the difference is the center-out difference. B) Polar histogram of angle differences from all pairwise comparisons between place cell maps calculated from single-unit data. C) Polar histogram of center-out angle differences from all pairwise comparisons between place cell maps calculated from calcium imaging data.

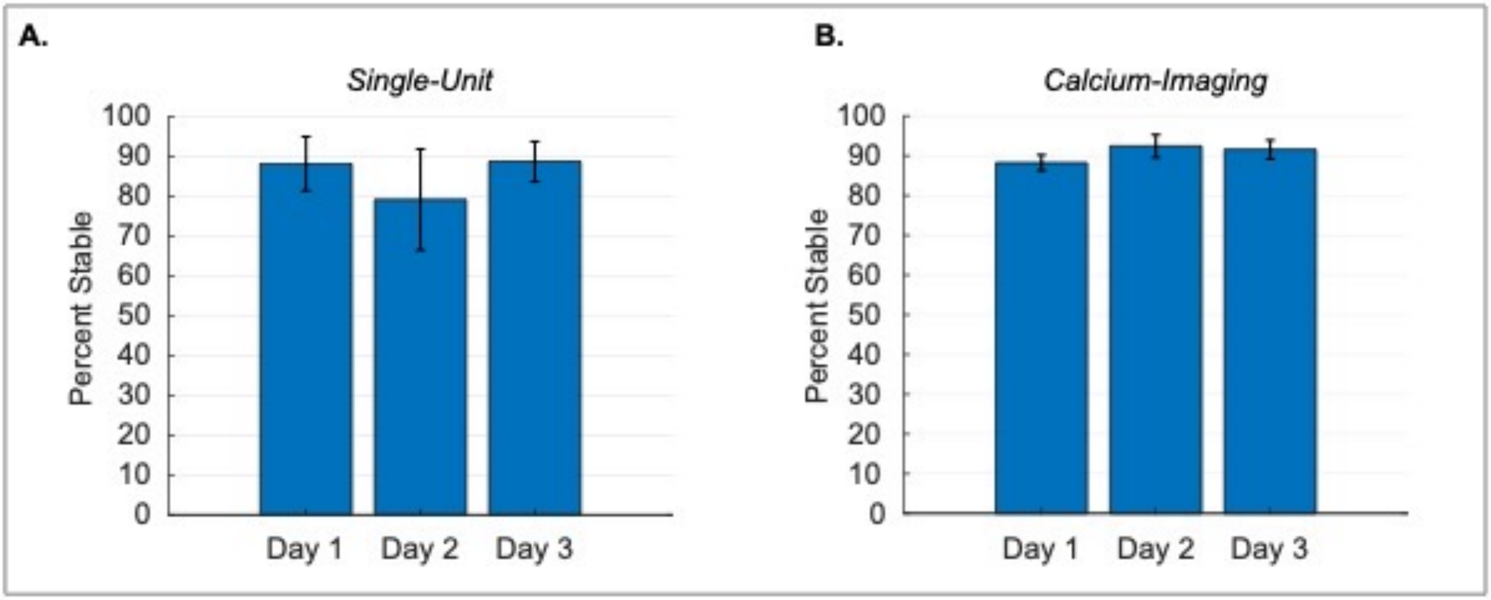

Figure S3. Similar proportions of cells with distinct remapping properties are present during reorientation independent of recording methiod. A) Proportion of stable cells from single-unit data. B) Proportion of stable cells from calcium-imaging data.

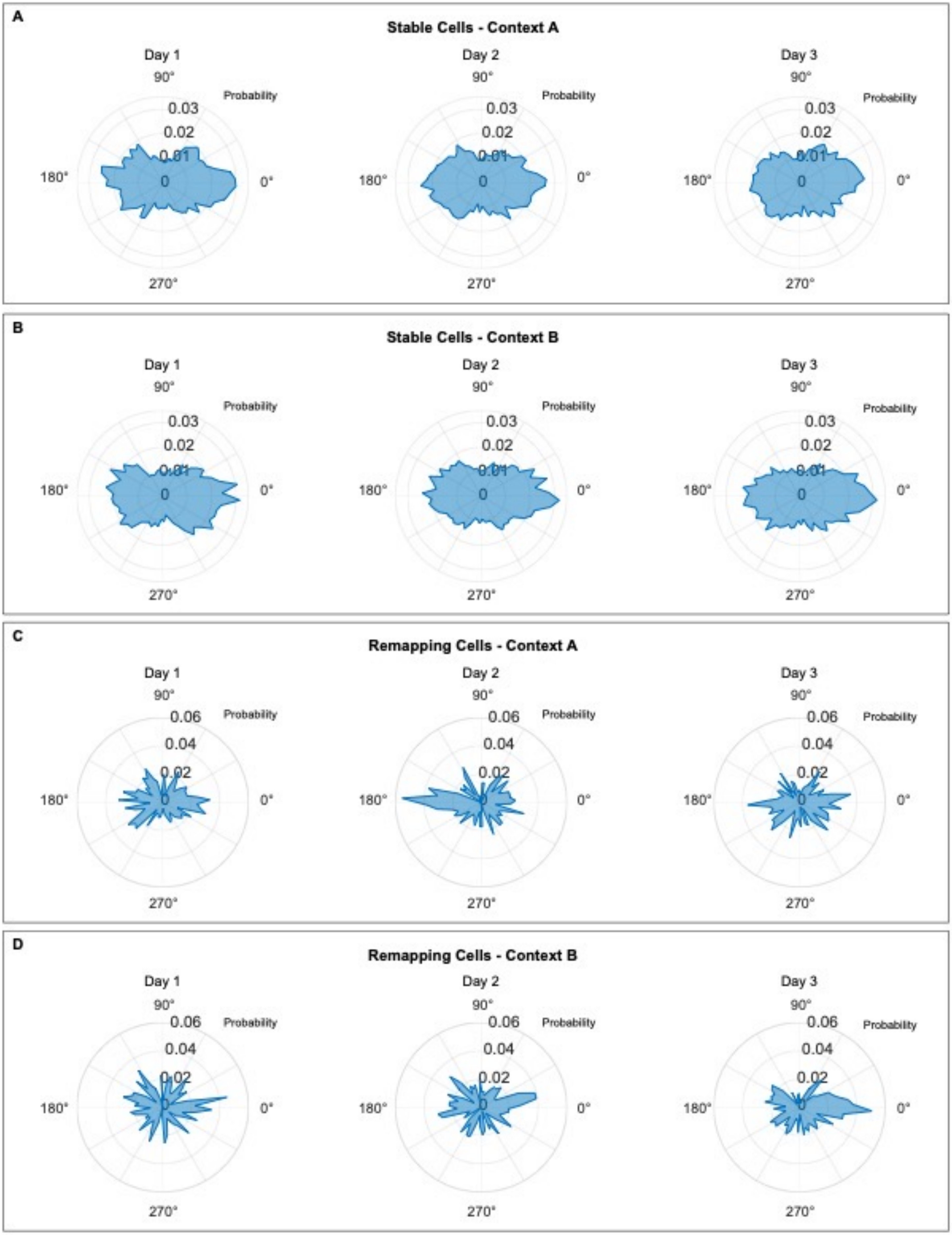

Figure S4. Polar histogram of center-out angle differences from pairwise comparisons generated in each context for stable and remapping cells. A-B) Center-out angle differences of stable cells in context A (A) and context B (B). C-D) Center-out angle differences of remapping cells in context A (C) and context B (D).

Figure S4

Table S3

Predictor	df_{Num}	df_{Den}	*Epsilon*	SS_{Num}	SS_{Den}	*F*	η^2_g	*p*
F. Sensitivity	1.00	2018.00		0.00	0.00	0.00	<.001	.955
Day	2.00	2018.00		0.00	0.00	0.01	<.001	.995
Context	1.00	2018.00		0.00	0.00	0.00	<.001	>.999
Rotation	2.89	5827.38	0.96	11.31	291.37	78.27***	.019	<.001
F. sensitivity x Day	2.00	2018.00		0.00	0.00	0.00	<.001	.999
F. Sensitivity x Context	1.00	2018.00		0.00	0.00	0.00	<.001	.997
Day x Context	2.00	2018.00		0.00	0.00	0.00	<.001	.998
F. Sensitivity x Rotation	2.89	5827.38	0.96	0.27	291.37	1.85	<.001	.138
Day x Rotation	5.78	5827.38	0.96	0.55	291.37	1.90	<.001	.080
Context x Rotation	2.92	5884.64	0.97	0.45	294.23	3.11*	<.001	.027
F. Sensitivity x Day x Context	2.00	2018.00		0.00	0.00	0.00	<.001	>.999
F. Sensitivity x Day x Rotation	5.78	5827.38	0.96	0.55	291.37	1.91	<.001	.079
F. Sensitivity x Context x Rotation	2.92	5884.64	0.97	0.09	294.23	0.59	<.001	.619
Day x Context x Rotation	5.83	5884.64	0.97	0.83	294.23	2.86**	.001	.009
F. Sensitivity x Day x Context x Rotation	5.83	5884.64	0.97	0.20	294.23	0.70	<.001	.645

Table S3. 4- way repeated measures ANOVA with Context (A or B) and Rotation (0°, 90°, 180°, 270°) as within-cells' factors, and F. Sensitivity (Feature-Sensitive or Feature-Insensitive) and Day (Day 1 to 3) as between factors. df_{Num} indicates degrees of freedom numerator. df_{Den} indicates degrees of freedom denominator. Epsilon indicates Greenhouse-Geisser multiplier for degrees of freedom, p-values and degrees of freedom in the table incorporate this correction. SS_{Num} indicates sum of squares numerator. SS_{Den} indicates sum of squares denominator. η^2_g indicates generalized eta-squared. * p≤ .05, ** p≤.01, ***p≤.001.
